# Amplitude modulating frequency overrides carrier frequency in tACS‐induced phosphene percept

**DOI:** 10.1002/hbm.26111

**Published:** 2022-10-17

**Authors:** Che‐Yi Hsu, Tzu‐Ling Liu, Dong‐Han Lee, Ding‐Ruey Yeh, Yan‐Hsun Chen, Wei‐Kuang Liang, Chi‐Hung Juan

**Affiliations:** ^1^ Institute of Cognitive Neuroscience, College of Health Sciences and Technology National Central University Taoyuan Taiwan; ^2^ Cognitive Intelligence and Precision Healthcare Research Center National Central University Taoyuan Taiwan; ^3^ Department of Psychology Kaohsiung Medical University Kaohsiung Taiwan

**Keywords:** amplitude‐modulated (AM) stimulation, Holo‐Hilbert spectral analysis (HHSA), phosphene, transcranial alternating current stimulation (tACS), visual awareness

## Abstract

The amplitude modulated (AM) neural oscillation is an essential feature of neural dynamics to coordinate distant brain areas. The AM transcranial alternating current stimulation (tACS) has recently been adopted to examine various cognitive functions, but its neural mechanism remains unclear. The current study utilized the phosphene phenomenon to investigate whether, in an AM‐tACS, the AM frequency could modulate or even override the carrier frequency in phosphene percept. We measured the phosphene threshold and the perceived flash rate/pattern from 12 human subjects (four females, aged from 20–44 years old) under tACS that paired carrier waves (10, 14, 18, 22 Hz) with different envelope conditions (0, 2, 4 Hz) over the mid‐occipital and left facial areas. We also examined the phosphene source by adopting a high‐density stimulation montage. Our results revealed that (1) phosphene threshold was higher for AM‐tACS than sinusoidal tACS and demonstrated different carrier frequency functions in two stimulation montages. (2) AM‐tACS slowed down the phosphene flashing and abolished the relation between the carrier frequency and flash percept in sinusoidal tACS. This effect was independent of the intensity change of the stimulation. (3) Left facial stimulation elicited phosphene in the upper‐left visual field, while occipital stimulation elicited equally distributed phosphene. (4) The near‐eye electrodermal activity (EDA) measured under the threshold‐level occipital tACS was greater than the lowest power sufficient to elicit retinal phosphene. Our results show that AM frequency may override the carrier frequency and determine the perceived flashing frequency of AM‐tACS‐induced phosphene.

## Graphical abstract

The current study examined the effect of amplitude‐modulated tACS on phosphene perception. The results showed that the amplitude‐modulating frequency increased the phosphene threshold and overrode the carrier frequency by changing the frequency function of flashing perception. This study, therefore, suggests amplitude modulation as a critical neural cross‐frequency‐coupling format in visual perception.

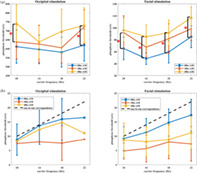

## 1 INTRODUCTION

Dynamical neural oscillations are essential for adaptive animal behaviors (Buzsáki & Mizuseki, 2014; Clarke et al., 2015). These brain oscillations are not sustained sinusoidal waves but complex spatial/temporal coordination between rhythms in the form of cross‐frequency coupling (CFC) (Buzsáki, 2006; Jensen & Colgin, 2007). Amplitude modulation (AM) is one of the CFC patterns, in which the amplitude of a faster carrier frequency (*f*_*c*_) and the phase of a slower frequency (*f*_am_) couple in such a way that the temporal change of *f*_*c*_ amplitude (that is, the envelope of *f*_*c*_) is in the frequency of *f*_am_ (please see Appendix S1: Supplementary 1, or figure 1 in Jensen & Colgin, 2007 for example). Numerous studies have indicated the importance of AM in brain activation related to fundamental cognitive functions (Canolty et al., 2006; Canolty & Knight, 2010; Fiebelkorn et al., 2018; Fries, 2005; Giraud & Poeppel, 2012; Hyafil et al., 2015; Liang et al., 2021; Palva & Palva, 2012; Salinas & Sejnowski, 2001; Siebenhühner et al., 2020; Siegel et al., 2012; Singer, 1999). Recently, people are getting aware of the utility of using AM transcranial alternating current stimulation (AM‐tACS) to modulate various cognitive functions (Alekseichuk et al., 2016; Lara et al., 2018; Riddle et al., 2022; Riddle & Frohlich, 2021; Turi et al., 2020). However, the mechanism of how AM‐tACS implicates neural activity is still unknown because not much neural evidence has been offered, and the stimulated areas usually did not provide an immediate sensory or motor output.

It is recently suggested that sensory stimulation in AM format, such as the visual flicker, can entrain the human visual system accordingly (Juan et al., 2021; Nguyen et al., 2019). Intriguingly, the authors found that the steady‐state visual evoked potentials (SSVEPs) demonstrated a 2–4 Hz AM effect even when participants were stimulated with non‐AM flicker. This result indicates an intrinsic AM characteristic in the visual system and suggests the visual system as a suitable model for verifying the neural mechanism of AM‐tACS. The current study, therefore, investigates the neural response to AM‐tACS with the phosphene paradigm, which indicates the light perception without light stimulation (Brindley & Lewin, 1968). Phosphene is perceived as repetitive flickering and is usually considered a side effect in tACS procedure (Fertonani et al., 2015). Previous studies have shown that percept to tACS‐induced phosphene (tACS‐phosphene) can be frequency‐dependent. In the study by Kanai and colleagues (Kanai et al., 2008), phosphene threshold (PT) was lowest around the alpha band in a dark room and switched to beta band in a well‐lit one (see Evans et al., 2019; Turi et al., 2013 for similar results). Besides, the percept of flickering speed changed according to the tACS frequency as well (Evans et al., 2019). These observations suggest that phosphene is sensitive to the oscillatory property of stimulation, which makes it an ideal tool for investigating the effect of AM‐tACS on visual percept.

One recent study using a simulated cortex model (Negahbani et al., 2018) suggested that during AM‐tACS, cortical activation would be phase‐locked to the enveloped AM frequency, and a higher intensity was needed to reach a phase‐locking level equivalent to sinusoidal stimulation. A later study by Thiele and colleagues (Thiele et al., 2021) supported the simulation result by observing AM‐tACS‐induced phosphene in human subjects. However, the original purpose of their study was to avoid tACS‐induced phosphene during stimulation and therefore the selected carrier frequencies were far higher than the visible range (>50 Hz). It was thus difficult to infer the AM effect due to lacking visible response. Moreover, because both studies compared thresholds between AM‐tACS (*f*_am_ + *f*_*c*_) and non‐AM‐tACS of modulating frequency (*f*_am_) rather than the carrier frequency (*f*_*c*_), the results could only demonstrate the AM effect in a very limited frequency range without observing the carrier frequency function and its interaction with AM frequency. The current study, therefore, adopts carrier frequencies within the visible range (10–22 Hz) based on previous studies (Evans et al., 2019; Kanai et al., 2008; Turi et al., 2013) to pair with slower AM frequencies (2–4 Hz). Despite the phosphene threshold, we collect the rating of flashing frequency and ask participants to draw phosphene patterns to further understand AM and carrier frequency effects in different aspects of the phosphene. It is expected that carrier frequency would affect both threshold and flashing frequency rating, and the AM would raise the threshold and might dominate the phosphene percept by slowing the perceived flashing.

Further verify the source of AM effect, the generator of phosphene would be a critical issue. Kanai and colleagues (Kanai et al., 2008) proposed the tACS‐phosphene generator to be the anterior part of the visual cortex. However, some questioned whether tACS can directly influence the cortex (Schwiedrzik, 2009) because it was suggested that most of the current was found to be lost through shunting from skin, skull, and soft tissues (Asamoah et al., 2019; Huang et al., 2017; Vöröslakos et al., 2018). Supporting the suspicion, later studies using phosphene measurements and simulation models suggested that the shunted current might have elicited phosphene via reaching the retina (Evans et al., 2019; Kar & Krekelberg, 2012; Laakso & Hirata, 2013; Paulus, 2010; Schutter & Hortensius, 2010). Nevertheless, the cortical contribution during tACS‐induced phosphene cannot be excluded completely for the following reasons. Firstly, the conventional large rubber electrodes usually elicit a wide‐range current distribution that inevitably affects the retina (Indahlastari et al., 2018; Mehta et al., 2015; Vöröslakos et al., 2018). This causes difficulty in dissociating phosphene that is generated from the cortex or the retina (Paulus, 2010; Schutter & Hortensius, 2010). Secondly, computation model (Laakso & Hirata, 2013) suggested that the amount of current that can penetrate the orbit, even under the wide‐spreading distribution, is extremely limited. It was also proposed that changing the montage arrangement may limit the current distribution and decrease current flow to the retina (Laakso & Hirata, 2013; Mehta et al., 2015). Finally, the activation characteristic was found to differ between occipital and frontal stimulation montages (Evans et al., 2019). This finding implies that the phosphene perceived under occipital stimulation may have resulted from mixed neural generators. To address these issues, our study applied more focal current distributions with a high‐density stimulation system. We expect to observe a clearer montage effect that differentiates between phosphene from the retina and the primary visual cortex.

To monitor the current flow through the skin to the eyes, we measured the near‐eye electrodermal activity (EDA) as an index of the epidermis potential change. We collected the near‐eye EDA and calculated the power of applied frequency under each participant's phosphene threshold (PT) intensities. We compared the power between occipital and forehead/upper‐cheek stimulations. The EDA under facial PT stimulation is defined as the “threshold EDA” of eliciting phosphene from the retina. If the EDA recorded during occipital PT stimulation was higher than the “threshold EDA”, it indicates that the leaked current from the weakest occipital montage was enough to elicit a retinal response, and the retina may also contribute to phosphene generation. In contrast, if the EDA under occipital PT stimulation is lower than the threshold EDA, the phosphene is more likely to generate from the visual cortex.

The current study aims to delineate the neural mechanism of amplitude‐modulated tACS and hopes to bridge the neural activation with visual perception by examining the relation between AM‐tACS properties and phosphene perception. To verify the source of the AM‐tACS phosphene, we adopt focal stimulation montages to overcome the problem of mixing contributions from the retina and visual cortex in previous studies.

## 2 METHODS

### 2.1 Participants

Twelve subjects were recruited from the National Central University, Taiwan. The subject number is based on calculation of G*Power (repeated measured within‐factor ANOVA, *α* error = 0.05, medium effect size partial *η* ^2^ = 0.06, number of measurements = 24, correlation among repeated measure = 0.5, Nonsphericity correction = 1). All subjects had normal or corrected‐to normal vision and no history of neuronal diseases. The mean age of subjects was 27 years old (range from 20 to 44), and four were female. All subjects were informed of the experiment's procedure, and they agreed to and signed the consent form approved by the Institutional Review Board of the Chang‐Gung Memorial Hospital.

### 2.2 Experimental design and stimulation parameters

The current study adopted a within‐subject design, with factors of stimulation montage (facial or occipital montage), carrier frequency (10, 14, 18, and 22 Hz), and AM frequency (0, 2, and 4; 0 Hz denotes no amplitude modulation was applied on the carrier frequency). The frequency selection was based on previous studies (Evans et al., 2019; Kanai et al., 2008; Turi et al., 2013) and our pilot experiment (see Appendix S1: Supplementary 2 for the pilot result). We chose the carrier frequencies that cover the most sensitive alpha and beta bands, and AM frequencies that participants reported seeing phosphene most often in the pilot experiment. Based on these three factors, each participant received 24 stimulation conditions, including 12 different carrier‐AM frequency combinations applied on either left facial or occipital area.

**FIGURE 1:**
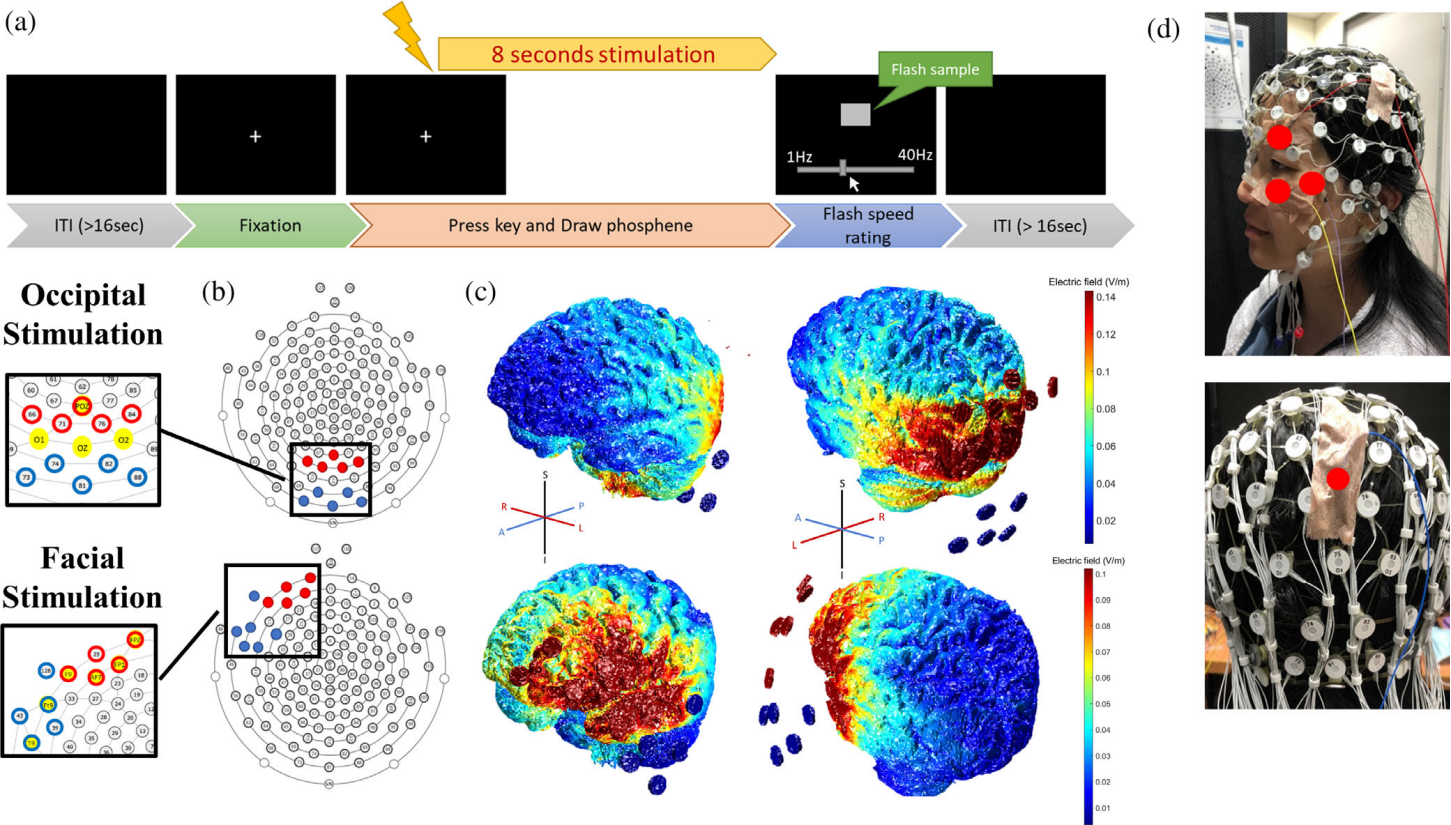
(a) Trial structure of the task. Participants fixated their eyes on the center of the screen. Once the stimulation started, they pressed the space key and drew the phosphene pattern if they saw one; otherwise, they pressed the key to skip the trial. In the flash speed rating scene, they moved the sliding bar by mouse to change the flickering frequency of the sample to reproduce the phosphene they saw and pressed the key again to finish the trial. (b) Location of electrodes selected to conduct tACS. The facial stimulation montage covered the left forehead and upper cheek (channel number grouped in polarity: 21, 22, 25, 26, 32 and 128, 38, 39, 43, 44 in the EGI system) and the occipital montage covered the middle area of the occipital scalp (channel numbers grouped in polarity: 66, 71, 72, 76, 84 and 73, 74, 82, 88, 81 in the EGI system). (c) the illustration of the simulated current distribution is based on the electrode layout of (b) under the intensity of 1 mA. The estimated current of the left facial stimulation montage is strongest over the left frontal pole and is expected to stimulate the eye (not illustrated here). The estimated current of occipital stimulation montage is strongest around primary visual cortices on the bilateral occipital lobe. The intensity of the estimated current is denoted by the color bars. The simulations were done with the ROAST software (Huang et al., 2019). (d) Demonstration of the location of EDA electrodes over skin near eyes (upper) and occipital (lower) stimulation area. The recording electrodes were fixed with medical tape and marked with red dots. Only signals from the above‐eyebrow electrode were analyzed because it recorded the most significant signals than the two other near‐eye electrodes

The AM stimulation waveforms were generated with MATLAB (MathWorks, Natick, MA) with the following equation:

$$y\left(t\right)=\frac{1}{2}\left(\sin\left(2\pi f_{\mathrm{am}}t-\frac{\pi }{2}\;\right)+1\right)\sin\left(2\pi f_{c}t\right)$$

where *f*_am_ indicated the envelope or modulation frequency, and *f*_*c*_ indicated the carrier frequency. The stimulation was conducted with the Geodesic Transcranial Electrical Neuromodulation (GTEN) Planning Module (Electrical Geodesive Inc.; EGI, Eugene, OR, USA) through 128‐channel elastic cap. Ten electrodes (five anodes and five cathodes. The selected channel numbers on the EGI cap are listed in Figure 1. Also see Appendix S1: Supplementary 3 for the corresponding list between EGI the 10‐10 system) were selected to deliver current stimulation in occipital and left facial regions, respectively. The diameter of each electrode was 8 mm, which makes the area around 0.5 cm^2^ for each electrode. The facial stimulation montage covered the left forehead and upper cheek, which aimed to stimulate the left eye. The occipital montage covered the middle area of the occipital scalp which aimed for the primary visual cortex. The impedance of all electrodes was lower than 50 kΩ during stimulation. To ensure the range of stimulation fits our aim, we estimated the electric field distribution of 8 out of 12 participants (who had a T1 structure image) with the ROAST software (Huang et al., 2019).

### 2.3 Experimental procedures

Before the formal experiment, participants were informed about the phenomenon of phosphene as well as the individual difference in phosphene sensitivity to minimize the possibility of guessing and expectation. Participants first received the highest intensity (1000 uA) stimulation to see if he/she could perceive phosphene and get familiar with the possible tactile sensation caused by the stimulation. Participants who could not perceive phosphene with 1 mA stimulation on the occipital area were excluded from the experiment. In the formal experiment, participants came for two experimental sessions with at least a one‐week gap. In each session, participants were stimulated with either occipital or left facial montage. The order of occipital and left facial sessions was counterbalanced across participants. During each montage session, participants were tested with 12 stimulation waveforms in random order to find the phosphene threshold. Each condition took about 6–7 trials to find the threshold intensity. Each session took 70–80 stimulation trials to finish. For more detailed information about the threshold measurement method please refer to Appendix S1: Supplementary 4.

During the experiment, participants were seated in a dimly lit room (0.25 cd/m^2^). Observation distance was fixed with a chin rest at 60 cm from a 24‐inch, 120 Hz refresh rate LCD monitor. The task was programmed in Matlab (R2015b) using Psychtoolbox‐3 (PTB‐3) (Brainard, 1997; Pelli, 1997; Kleiner et al., 2007). Participants fixated on a 0.7 × 0.7 visual degree white fixation point displayed in the center of a full‐screen black background. Figure 1a demonstrates the visualized trial structure. Each trial started with a fixation in the center of the screen, followed by a tACS that lasted for 8 s, including a 1‐s ramp‐up and ramp‐down. Participants were asked to fixate their eyes on the fixation and to press the space key when perceiving phosphene. They then used a mouse to draw the perceived phosphene as spreading dots during the stimulation. The coordinates of the spreading dots were recorded for pattern analysis. Participants then proceeded to the next frame to report the flashing rate of the phosphene. A horizontal sliding bar appeared on the lower side of the screen. When they moved the sliding bar to a frequency between 1 Hz (left pole) and 40 Hz (right pole), a square appeared in the center of the screen and flashed at the frequency indicated by the sliding bar. Participants were required to adjust the flashing frequency of the square with the sliding bar to reproduce the flashing frequency of their phosphene. Finally, they pressed the space key to enter the subsequent trial if no phosphene was detected or when the report was done.

### 2.4 EDA recording and analysis

The near‐eye EDA was recorded with the Biopac MP36 (Biopac Systems) at a 1000 Hz sampling rate. Signals were recorded with 3 Ag/CL electrodes contacted on the left side, supra‐eyebrow, and lower side around the left eye (upper Figure 1d). The electrodes were referenced to the left lateral malleolus and were fixed with conductive gel and medical tape to keep the impedance under 50 kΩ. For each stimulation condition, we recorded the last trial in which the participant reported seeing the phosphene (the “just above threshold” intensity) for analysis. We analyzed the EDA signals from the supra‐eyebrow electrode because it recorded the greatest potential change among the three near‐eye electrodes and was most likely to pick up currents going to the retina. The continuous signals were separated into epochs with up to 6000 data points covering the stimulation duration, excluding the ramp‐up and ramp‐down stages. Due to the nonlinearity of the stimulation waves, we adopted the Holo‐Hilbert Spectral Analysis (HHSA, Huang et al., 2016) to decompose the EDA signal and obtain power values of AM and carrier frequencies (See Appendix S1: Supplementary 5 for details).

### 2.5 Statistical analysis of behavioral data

The threshold intensity, mean flash frequency rating, and the mean coordinates of the phosphene drawings were calculated for each condition. A three‐way repeated‐measures ANOVA with factors of montage (occipital vs. left face), AM frequency (0, 2, and 4 Hz AM), and carrier frequency (10, 14, 18, and 22 Hz) was conducted to the phosphene thresholds, flashing frequency ratings, and mean coordinates of phosphene drawing. The normalized EDA power of each stimulation condition was calculated based on the HHSA methods described in Appendix S1: Supplementary 5. For each montage, the power values were averaged across all AM and carrier conditions. The mean power values were then entered a paired *t*‐test to examine the power difference between occipital and left facial stimulations.

## 3 RESULTS

Three sets of repeated measured ANOVA and one paired *t*‐test were conducted in the analysis. The results are summarized below in Table 1.

**TABLE 1:** The summary table of statistical results

	Statistic results
Threshold intensity	
*Montage (MT)*	*F*_(1,11)_ = 169.39, *p <* 0.001, *η*_p_^2^ = 0.94
*AM frequency (AM)*	*F*_(2,22)_ = 12.55, *p <* 0.001, *η*_p_^2^ = 0.53
*Carrier frequency (Fc)*	*F*_(3,33)_ = 10.57, *p* < 0.001, *η*_p_^2^ = 0.49
*MT* × *AM*	*F*_(2,22)_ = 3.58, *p* = 0.045, *η*_p_^2^ = 0.25
*MT* × *AM* × *Fc*	*F*_(6,66)_ = 2.75, *p* = 0.019, *η*_p_^2^ = 0.2
Flash frequency rating	
*AM*	*F*_(2,14)_ = 11.7, *p* = 0.001, *η*_p_^2^ = 0.63
*Fc*	*F*_(3,21)_ = 10.17, *p* < 0.001, *η*_p_^2^ = 0.59
*AM* × *Fc*	*F*_(6,42)_ = 4.04, *p* = 0.05, *η*_p_^2^ = 0.37
Mean coordinates	
*MT*	*X*: *F*_(1,7)_ = 58.37, *p <* 0.001, *η*_p_^2^ = 0.89
	*Y*: *F*_(1,7)_ = 19.96, *p* = 0.003, *η*_p_^2^ = 0.74
Near‐eye EDA power (paired *t*‐test, occipital—left facial)	
*MT*	AM power: *t*_10_ = 3.18, *p* = 0.01, Cohen's d = 0.8
	Carrier power: *t*_10_ = 3.44, *p* = 0.006, Cohen's d = 1.4

*Note*: The degree of freedom was different in the mean coordinates analysis due to conditions containing no response. *η*_p_^2^: partial eta squared.

### 3.1 Phosphene threshold

**FIGURE 2:**
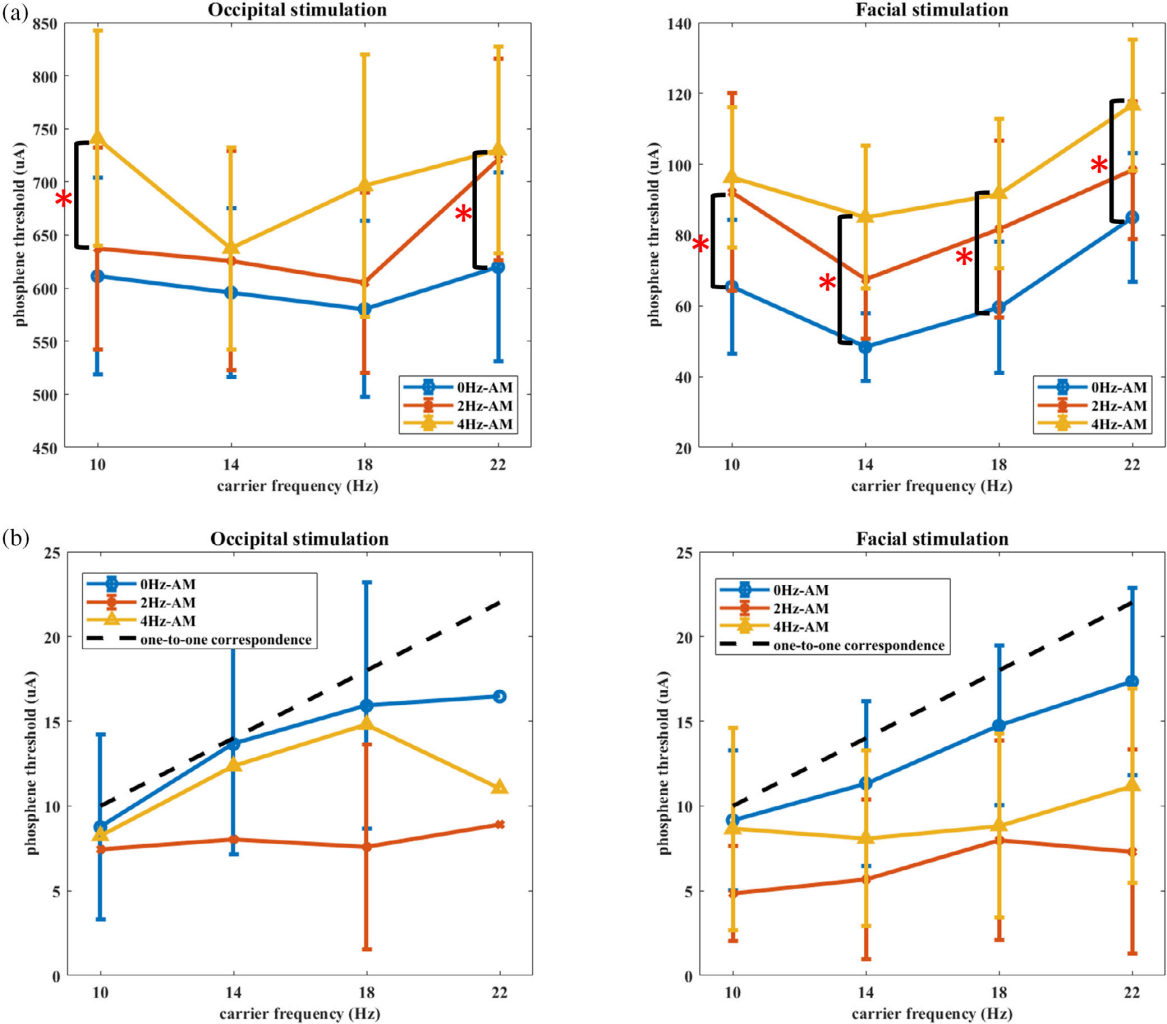
(a) Illustration of the mean phosphene thresholds (PT) with 95% CI (indicated by error bars) of occipital (left) and facial (right) montages. Note that the y‐axis scale differs between montages. In both montages, PTs were lower for 14 Hz and 18 Hz compared to 10 and 22 Hz. For the AM frequency effect, PT was highest for 4 Hz AM and lowest for 0 Hz AM (non‐AM) condition. (b) Illustration of the mean flashing frequency rating during occipital (left) and facial (right) stimulation (95% CI marked with error bars). The black dashed line indicates the hypothesized one‐to‐one (*y* = *x*) correspondence in which participants rated exactly the given frequency. Ratings in 0 Hz AM (blue lines) condition increase as the given carrier frequency increases. Ratings in 2 Hz (orange) and 4 Hz (yellow) AM conditions showed slower ratings and flatter trends compared to 0 Hz AM condition.

The three‐way ANOVA on the phosphene threshold (PT) shows significant main effect of montage (*F*_[1,11]_ = 169.39, *p <* 0.001, *η*_p_^2^ = 0.94*)*, indicating higher PT for occipital (mean value with 95% CI: 650.14 ± 99.00) than left facial montage (82.29 ± 15.63). As illustrated in Figure 2a, main effect of AM frequency was significant (*F*_[2,22]_ = 12.55, *p <* 0.001, *η*_p_^2^ = 0.53). Paired comparisons with Bonferroni correction showed higher PT for 4 Hz AM (399.27 ± 60.41) than both 2 Hz AM (366.15 ± 53.78, *p* = 0.035) and 0 Hz AM (333.23 ± 49.52, *p* < 0.004). The main effect of carrier frequency was significant (*F*_[3,33]_ = 10.57, *p* < 0.001, *η*_p_^2^ = 0.49). Following comparisons showed that PT for 14 Hz (343.33 ± 55.93) was significantly lower than 10 Hz (373.89 ± 56.81) and 22 Hz (395.21 ± 41.29), and the PT for 18 Hz (352.43 ± 51.45) was lower than 22 Hz. The contrast analysis on the carrier frequencies showed a significant quadratic trend (*F*_[1,11]_ = 34.57, *p <* 0.001, *η*_p_^2^ = 0.76).

The results also revealed a two‐way interaction between montage and AM frequency (*F*_[2,22]_ = 3.58, *p* = 0.045, *η*_p_^2^ = 0.25) and a significant three‐way interaction among montage, AM frequency, and carrier frequency (*F*_[6,66]_ = 2.75, *p* = 0.019, *η*_p_^2^ = 0.2). Further analyses showed unequal AM effect in two montages. In the facial montage, AM frequency effect was significant in all carrier frequencies (10 Hz: *F*_(2,10)_ = 7.45, *p* = 0.01, *η*_p_^2^ = 0.6; 14 Hz: *F*_(2,10)_ = 6.54, *p* = 0.015, *η*_p_^2^ = 0.57; 18 Hz: *F*_(2,10)_ = 13.77, *p* = 0.001, *η*_p_^2^ = 0.73; 22 Hz: *F*_(2,10)_ = 4.66, *p* = 0.037, *η*_p_^2^ = 0.48), while for the occipital montage, the AM frequency effect was only found in the 10 Hz (*F*_(2,10)_ = 4.36, *p* = 0.04, *η*_p_^2^ = 0.47) and 22 Hz (*F*_(2,10)_ = 5.9, *p* = 0.02, *η*_p_^2^ = 0.54) carrier frequencies.

### 3.2 Flash frequency rating

The mean flash ratings of each stimulation condition are illustrated in Figure 2b. The three‐way ANOVA on the flash frequency rating revealed main effect of AM frequency (*F*_[2,14]_ = 11.7, *p* = 0.001, *η*_p_^2^ = 0.63). Subsequent comparisons showed that flash was perceived faster for 0 Hz AM (13.76 ± 5.4) than 2 Hz (5.51 ± 5.18, *p* = 0.02) and 4 Hz (8.63 ± 5.33, *p* = 0.045) AM. Main effect of carrier frequency was also significant (*F*_[3,21]_ = 10.17, *p* < 0.001, *η*_p_^2^ = 0.59). Subsequent comparisons showed faster ratings for 22 Hz (11.5 ± 5.8) and 18 Hz (10.85 ± 5.59) than 10 Hz (6.35 ± 3.25), but not for 14 Hz (8.5 ± 4.87). Contrast analysis showed a linear trend of carrier frequency (*F*_[1,7]_ = 20.02, *p* = 0.003, *η*_p_^2^ = 0.74) that the ratings increased as a function of carrier frequency. We also found a marginal interaction between AM frequency and carrier frequency (*F*_[6,42]_ = 4.04, *p* = 0.05, *η*_p_^2^ = 0.37). Subsequent analysis showed that the carrier frequency effect was significant only in the 0 Hz AM condition (*F*_(3,5)_ = 9.9, *p* = 0.02, *η*_p_^2^ = 0.86 for 0 Hz AM; *F*_(3,5)_ = 1.2, *p* = 0.4, *η*_p_^2^ = 0.42 for 2 Hz AM; *F*_(3,5)_ = 1.9, *p* = 0.24, *η*_p_^2^ = 0.5 for 4 Hz AM). No montage effect or interactions with other factors were found in this analysis.

Based on the linear trend found in the carrier frequency main effect, we examined whether the subjective ratings of phosphene flashing reflected the real stimulated frequencies in each of the AM frequencies. Data were pooled across two montages and fitted to a simple linear regression model y=x, where *x* indicates the given carrier frequency and *y* indicates the participants' rating. The results showed that participants' ratings in the 0 Hz AM fitted well to the model (*F*_[1,93]_ = 8.04, *p* = 0.006, *R* ^2^ = 0.08, coefficient *B* = 0.67), but the model could not explained performance in 2 Hz AM (*F*_[1,90]_ = 0.57, *p* = 0.45, *R* ^2^ = 0.006, coefficient *B* = 0.18) and 4 Hz AM (*F*_[1,87]_ = 0.84, *p* = 0.36, *R* ^2^ = 0.01, coefficient *B* = 0.23) conditions.

### 3.3 Phosphene pattern analysis

**FIGURE 3:**
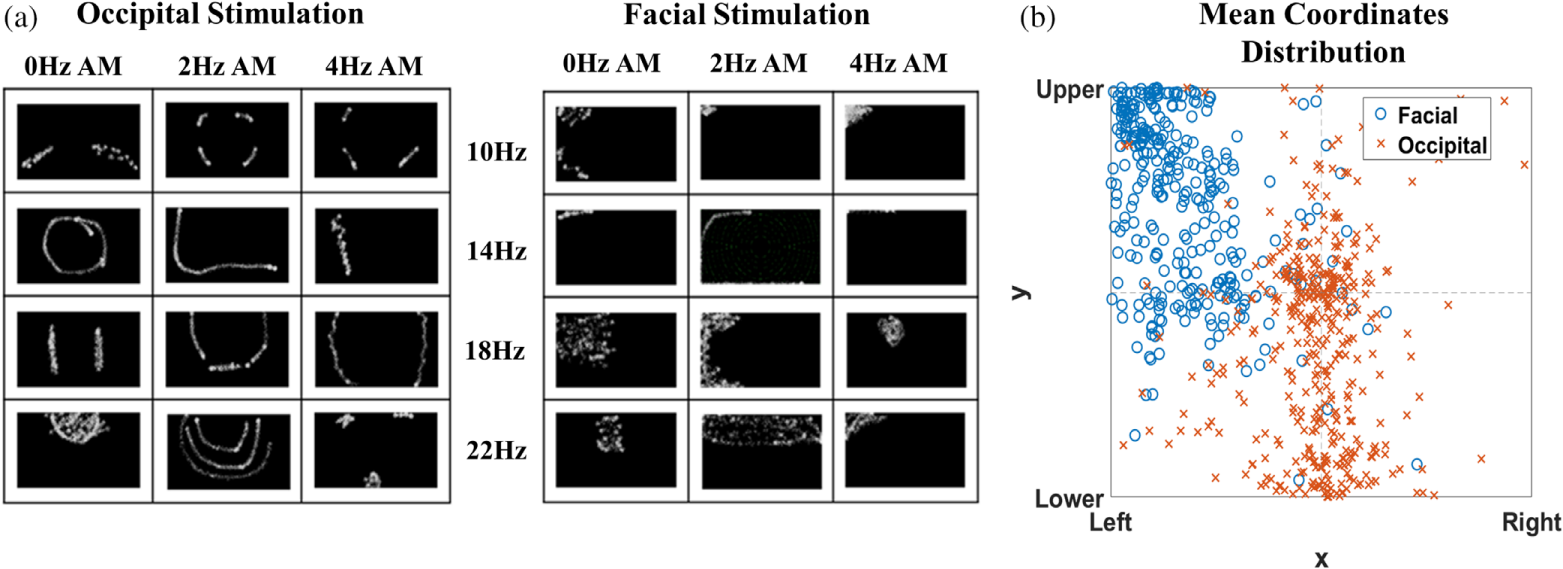
(a) An example of a phosphene drawing pattern of one participant under occipital (left) and left‐facial (right) stimulation. For the occipital stimulation, the flickering area is distributed symmetrically between the left and right halves of the screen. For the left‐facial stimulation, the phosphene focused on the upper‐left screen, representing that the participant saw flickering in the upper‐left visual field. The coordinates of each dot in one drawing were recorded for calculating the mean coordinate. (b) Distribution of mean coordinates calculated from each drawing response. As shown by the blue circles, most phosphenes of left facial stimulation focused on the left and upper areas. On the other hand, orange crosses represent that phosphene in occipital stimulation tended to distribute equally in the left and right halves of the screen

Figure 3a demonstrates examples of phosphene distribution (participant No.3) drawn under the threshold intensity stimulation (drawing of all participants are shown in Appendix S1: Supplementary 6). By visual observation, participants reported flashing over the upper‐left visual field under left facial stimulation, but the flashing was more symmetric under occipital stimulation. We illustrated the mean coordinates of all drawings in Figure 3b. A 2‐dimensional Kolmogorov–Smirnov test (Peacock, 1983; Muir, 2021) was conducted on the mean coordinates to examine whether the distributions from occipital and facial stimulation belonged to the same population. The result revealed a significant result (*D* = 0.85, *p* < 0.001) that the phosphene distribution differed between facial and occipital montages. We also conducted the three‐way ANOVA on the *x* and *y* values of the mean coordinates. Both examinations revealed significant montage effect (*x*‐values: *F*_(1,7)_ = 58.37, *p <* 0.001, *η*_p_^2^ = 0.89; *y*‐values: *F*_(1,7)_ = 19.96, *p* = 0.003, *η*_p_^2^ = 0.74). Compared to the phosphene perceived in occipital stimulation montage (*X* = 980.97 ± 41.23; *Y* = 680.28 ± 138.34; unit in pixel, with [960, 540] as the middle point. Based on Psychtoolbox, [0,0] is the upper‐left pole and [1920,1080] is the lower‐right pole. Therefore, the smaller number indicates left on the x‐axis and up on the y‐axis), phosphene in facial stimulation (*X* = 394.96 ± 197.6, *Y* = 311.85 ± 146.74) was more left‐lateralized and focused on the upper screen. None of the main effects of AM frequency, carrier frequency, and interactions were significant, showing that the stimulation condition did not affect the phosphene pattern except for the montage.

### 3.4 EDA results

**FIGURE 4:**
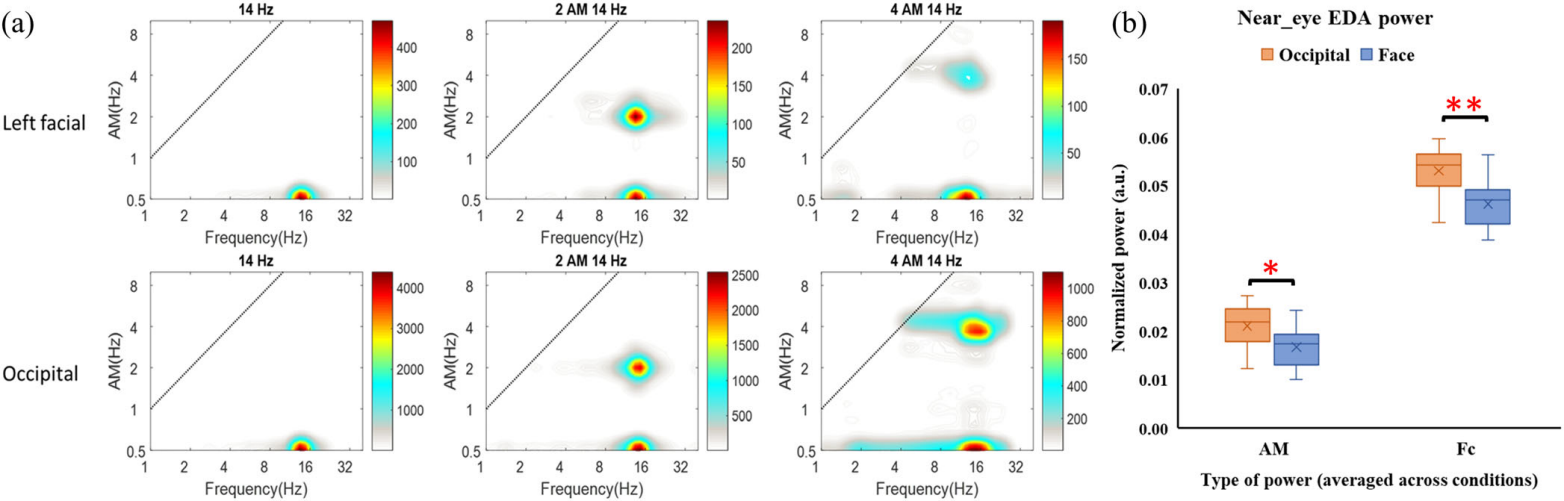
(a) An example of the spectral distribution result based on HHSA. The *x*‐axis indicates the carrier frequency, and the *y*‐axis indicates the AM frequency. The power value is denoted by color bars in an arbitrary unit. As shown by the figure, the power of skin potential correctly focused on the frequency range of given stimulation which is marked above each column. (b) the boxplot of normalized carrier and AM frequency power values (averaged across all frequency conditions) at the threshold levels of occipital and facial stimulations. As can be seen from the figure, the power of threshold‐level occipital stimulation is significantly higher than the threshold‐level facial stimulation in both AM and carrier frequency power. This result indicates that, even with a focal stimulation montage, the leaking current from occipital stimulation is still strong enough to trigger a retinal response and elicit phosphene

The EDA data from one participant were excluded from the analysis due to serious channel noise. Results of the HHSA (example illustrated in Figure 4a and complete spectrums illustrated in Appendix S1: Supplementary 7) revealed that the power of skin potential correctly focused on the range of given AM and carrier frequencies. The paired *t*‐test contrasting between occipital and left facial montages revealed that the carrier frequency power was significantly higher for the occipital (0.053 ± 0.003, arbitrary unit onormalized power) than facial (0.046 ± 0.003) stimulation (*t* = 3.44, *p* = 0.006, Cohen's d = 1.4), while similar result was found in AM frequency (*t* = 3.18, *p* = 0.01, Cohen's d = 0.8; 0.021 ± 0.003 for occipital montage; 0.017 ± 0.0002 for left‐facial montage). Please see Figure 4b for boxplots of participants' mean normalized power.

## 4 DISCUSSION

The current study examined how amplitude‐modulated current stimulation can affect the visual percept by comparing the phosphene percept under sinusoidal and AM‐tACS. To do this, we measured participants' phosphene threshold, phosphene flash frequency, and phosphene pattern when under the influence of AM‐tACS. To verify the source of the phosphene, we also recorded the near‐eye EDA to examine whether the current shunted from the skin during occipital stimulation can elicit a retinal response.

### 4.1 AM effect on phosphene threshold

The phosphene threshold results of the sinusoidal left facial stimulation showed the lowest threshold around 14 and 18 Hz and a higher threshold for 10 and 22 Hz, replicating the frequency effect under a lighted environment in previous studies (Kanai et al., 2008; Turi et al., 2013; Evans et al., 2019). The main effect of AM frequency indicated that compared to sinusoidal tACS, AM‐tACS needed higher intensity to elicit phosphene, which agrees with recent findings (Negahbani et al., 2018; Thiele et al., 2021). Because the threshold change in AM‐tACS was suggested to be a result of weaker intensity (Thiele et al., 2021), we selected the near‐eye EDAs under the same intensity and examined the effect of AM frequency (see Appendix S1: Supplementary 8 for detailed analysis). The result supported Thiele et al.'s (Thiele et al., 2021) argument by showing significantly greater power for 0 Hz AM than 2 or 4 Hz AM conditions, also greater power for 2 Hz AM than 4 Hz AM. Our result on the phosphene threshold, therefore, suggested that the AM effect on the phosphene threshold was mainly a result of a change in stimulation intensity.

### 4.2 AM effect on phosphene flashing frequency rating

The speed of phosphene flash had been reported in a previous study (e.g., Evans et al., 2019), but only in qualitative descriptions (e.g., “pulses”, “flashes”). The current study quantified the perceived flashing rate by asking the participant to reproduce the flashing speed of their phosphene with the sliding bar and flickering square. We demonstrated that the perceived phosphene correctly reflected the physical tACS frequency and fitted with the one‐to‐one “physical stimulation—psychological perception correspondence” model between 10–22 Hz. A slight rating decrement was found in the 22 Hz, which possibly reflected the difficulty of differentiating between high flash frequencies, or some might have reached their flicker fusion threshold (Davis, 1955). More importantly, the relation between given frequency and flash rating was abolished in the AM conditions that participants perceived generally slow flickering in 2 Hz and 4 Hz AM conditions regardless of the carrier frequency.

**FIGURE 5:**
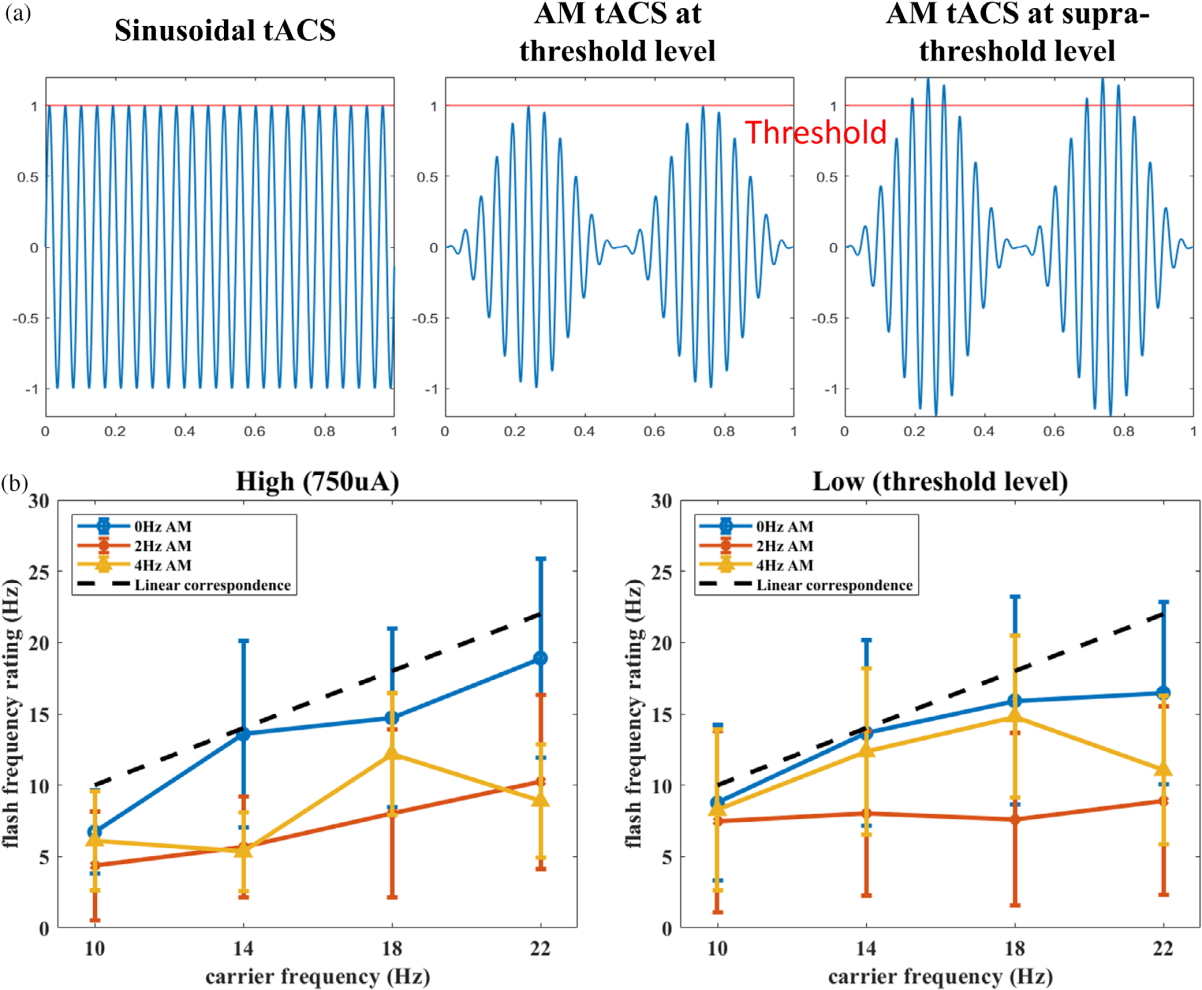
(a) The scheme of the hypothesized explanation for the AM effect on flash ratings. As shown in the figure, when given a sinusoidal tACS (left) the peaks of oscillation reached the threshold intensity (marked with a red horizontal line) and elicit a fast phosphene percept. When given an AM‐tACS at the threshold level (middle), the participant may perceive a slow flash because only the peak of the AM frequency can reach the threshold level. If this explains the AM effect, we infer that giving a supra‐threshold AM‐tACS (right) should accelerate the perceived flash because more peaks of the oscillation will reach the threshold intensity. (b) Illustrations of the result of flash frequency rating under supra‐threshold (left) and threshold (right) intensities. The mean ratings (with 95% CI indicated by error bars) frequency rating demonstrates that there is no significant change in the frequency rating between the two intensity levels. For statistical analysis, data of three participants were excluded due to too many missing values (more than two out of four carrier frequency levels in each AM frequency). An ANOVA adopting factors of intensity (supra‐threshold/threshold), AM frequency (0/2/4 Hz) and carrier frequency (10/14/18/22 Hz) revealed no intensity main effect, nor the interactions with intensity (all *p* values >0.2)

One possible explanation for this percept change lies in the waveform of the AM condition. As illustrated in Figure 5a, when tACS is given in sinusoidal waveform, the intensity reaches the threshold level multiple times and creates high‐frequency flashing. However, when stimulation is delivered in AM waveform, only the oscillation in the peak or trough of the envelope could reach the threshold level. Therefore, participants may have perceived slower flashing because the intensity was not strong enough to elicit phosphene most of the time. If this is the case, giving an AM‐tACS in a supra‐threshold intensity should increase the oscillations exceeding threshold levels and therefore increase the perceived flash frequency. We conducted a supplementary analysis that selected the flash frequency ratings under a supra‐threshold intensity (750 uA) and compared them with ratings of the lower threshold intensity. However, results (Figure 5b) revealed no intensity main effect or its interaction with other factors, suggesting that AM did not lower the flash frequency by decreasing the frequency carrier oscillation reached threshold intensity.

The question that the AM frequency seems to dominate the percept flashing frequency remains open to discussion. It has long been known that the cortical neurons in the primary auditory cortex encode auditory signals by phase‐locking to the signal envelope (the temporal envelope) (Liang et al., 2002). In the visual system, temporal envelope information was found to be encoded early in the cat V2 area (Shapley, 1998; Mareschal & Baker Jr., 1998). Our previous study (Nguyen et al., 2019) has also demonstrated that when entraining the visual system with an AM flicker, the AM frequency in SSVEP broadly modulated various frequencies rather than solely the given carrier frequency, suggesting that the human visual system also encodes AM information accordingly. On the other hand, a seminal study by Smith et al. (2002) demonstrated that temporal envelope determined speech reception, while the fine structure (the carrier frequency) defined pitch and location of a sound. It is thus suggested that human perception picks up both carrier and AM frequencies. If the visual system contains architecture similar to the auditory system, the AM frequency effect we found may be a result of neural phase‐locking to envelope frequency in the visual system. A previous study using simulated cortical neurons (Negahbani et al., 2018) has indicated that higher intensity created a stronger phase‐lock to the modulating frequency. We, therefore, consider that the perceived flash frequency may reflect the envelope frequency in which the cells are phase‐locking. When receiving a sinusoidal tACS, neurons are phase‐locked to the carrier frequency and generate fast flashing. When stimulated with AM‐tACS, neurons are phase‐locked to the AM frequency and the individual perceives slow flashing. Results from the current experiment are also in line with the recent proposal that the slow wave in the brain provides a “temporal receptive window” that binds multiple stimuli into one perceptual representation, and therefore plays a role in perceptual integration/segregation (Golesorkhi et al., 2021; Northoff & Huang, 2017; Wolff et al., 2022). Our results, combined with previous findings (Liang et al., 2002; Mareschal & Baker Jr., 1998; Negahbani et al., 2018; Shapley, 1998; Smith et al., 2002), thus suggest that the neural phase‐lock to envelope signals may be the underlying mechanism of perception formation.

### 4.3 Phosphene pattern

The phosphene patterns under PT intensity stimulation demonstrated a significant montage effect regardless of frequency. The occipital stimulation elicited phosphene on the upper or lower visual field, while some participants also reported phosphene on the central visual field. Kanai and colleagues (Kanai et al., 2008) proposed that the peripheral phosphene corresponded to current stimulation on the anterior part of the visual cortex on the medial wall. However, the phosphene may also reflect peripheral retinal activation. The conventional hypothesis from computational models suggests that the leaked current stimulated the orbit with a higher current over the peripheral than the central retina (Laakso & Hirata, 2013; Lorenz et al., 2019). Another “surface hypothesis” by Kanamaru and colleagues (Kanamaru et al., 2020) proposed that the current only stimulates the exposed eyeball rather than directly stimulating the retina. However, the surface hypothesis was built on facial stimulation, it is therefore unclear whether occipital stimulation follows the same model.

On the other hand, left‐facial stimulation elicited phosphene in the upper‐left visual field. This result is quite surprising if presuming the current penetrates the scalp and stimulates the retina underneath because the temporal retina of left eye receives images from the right visual field (Trobe, 2001). Previous studies also found ipsilateral phosphene with lateralized stimulations (Higuchi et al., 2017; Kanamaru et al., 2020; Turi et al., 2013), but the mechanism of phosphene generation was not in good consensus. The conventional hypothesis (Laakso & Hirata, 2013) proposed that current flows around the orbit stimulate not only the temporal but also the nasal part of the retina, which receives the image from the lateral visual field. However, it could not explain why phosphene did not appear in the central visual field if the temporal retina was also affected. In contrast, the surface hypothesis demonstrated that when eyes move left or right, the nasal retina of the left or right eye is exposed to the surface and receives stimulation directly (Kanamaru et al., 2020). However, participants in our study were asked to look to the front and should not expose the nasal retina. Although Kanamaru and colleagues (Kanamaru et al., 2020) suggested the “indirect stimulation” may affect from outside of the orbit, the mechanism remains to be explored.

### 4.4 EDA results and source of tACS‐induced phosphene

The near‐eye EDA analysis aims to examine whether the leaking current during occipital stimulation at PT level was enough to elicit retinal phosphene. Both AM and carrier frequency power during left‐facial‐PT stimulation was treated as the “threshold EDA,” (mean normalized power with 95% CI: 0.017 ± 0.002 for AM frequency; 0.046 ± 0.003 for carrier freqeuncy) and we found the EDA recorded during occipital‐PT stimulation (0.021 ± 0.003 for AM frequency; 0.053 ± 0.003 for carrier frequency) was higher than the threshold EDA. This result implies that the leaking current to the eye during occipital stimulation with its least intensity was strong enough to elicit a retinal response, which is in line with previous findings (Evans et al., 2019; Schwiedrzik, 2009; Paulus, 2010; Vöröslakos et al., 2018) to support retina as the phosphene generator of occipital tACS. Indahlastari and colleagues (Indahlastari et al., 2018) estimated the current density in occipital cortex and eye using Fpz‐Oz and T7‐T8 montages. Their result revealed a higher current density in the eye than in the visual cortex, also, the current density in the eye predicted participants' phosphene rating better than which in the visual cortex. Our simulation result on a subset of participants (for the results please see Appendix S1: Supplementary 9) obtained a similar result that the anterior cortical electric field was stronger during the occipital stimulation compared to the left‐facial stimulation. Furthermore, a study on in vivo animals suggested that it takes at least 1 V/m to change neural spiking (Vöröslakos et al., 2018). All simulated electric field values over visual cortex in our study were lower than 0.5 V/m, which indicates that occipital stimulation may not elicit cortical activation in the current study. Therefore, both our EDA and cortical simulation results provide evidence supporting retina as the source of tACS‐induced phosphene.

## 5 LIMITATIONS

The current study has two main limitations: (1) stimulation with high‐definition electrodes is usually arranged in a ring‐shape montage to limit the current distribution. However, we found it difficult to elicit phosphene with a ring‐shaped montage in the pilot study. Moreover, to the best of our knowledge, no study has ever elicited phosphene with a ring‐shaped montage. We, therefore, adopted the electric pad‐like montage that two groups of electrodes being the positive and negative polarities. To better limit the current distribution, we arranged both positive and negative electrodes on the occipital area. According to model simulation results (Laakso & Hirata, 2013), putting both polarities on the occipital scalp area can effectively decrease the total current flow to retina and create a relatively focal stimulation. (2) All trials in the current study were valid trials with stimulation. One may doubt whether participants may report false positives during the test. However, the stimulation was long in our study (8 s) and participants were asked to plot the phosphene area during stimulation. All these steps made participants respond only to stable and lasting flickering in the visual field and helped to avoid false positive response which is often observed in short visual presentation.

## 6 CONCLUSION

Results of the current study demonstrated that the amplitude modulation from a slow wave raised the phosphene threshold, and more importantly, dominated the subjective feeling of phosphene flashing. The dominance of AM frequency suggests that the neural phase‐locking to envelope information may play a critical role in perception formation. On the practical side, these results provide more flexible tACS parameters in inducing or avoiding phosphene during stimulation. For example, the low‐frequency stimulation (e.g., 2 Hz) usually does not induce phosphene, but participants may perceive phosphene in the form of 2 Hz AMtACS or vice versa. Finally, our study may provide further insight for visual prostheses applications such as the hand‐free support system for blind people (Kanamaru et al., 2020) or virtual/augmented reality techniques through retinal stimulation (Higuchi et al., 2017).

## AUTHOR CONTRIBUTIONS

Che‐Yi Hsu, Tzu‐Ling Liu, Yan‐Hsun Chen, and Chi‐Hung Juan designed the experiment. Dong‐Han Lee and Yan‐Hsun Chen programmed the experiment. Che‐Yi Hsu and Ding‐Ruey Yeh acquired the data which Che‐Yi Hsu, Tzu‐Ling Liu, and Wei‐Kuang Liang analyzed. Che‐Yi Hsu, Tzu‐Ling Liu, and Chi‐Hung Juan wrote the article.

## CONFLICT OF INTEREST

The authors declare that the research was conducted without any commercial or financial relationships that could be construed as a potential conflict of interest.

## ETHICS STATEMENT

The studies involving human participants were reviewed and approved by the Research Ethics Committee of Chang‐Gung Memorial Hospital. Furthermore, the patients/participants provided their written informed consent to participate in this study.

## Supporting information


Appendix S1:

## Data Availability

The raw data supporting the conclusions of this article are available from the corresponding author upon reasonable request.
