# Supplementary material for: Amplitude modulating frequency overrides carrier frequency in tACS‐induced phosphene percept
Source: Hum Brain Mapp. 2022 Oct 17;44(3):914–26. doi: 10.1002/hbm.26111 (PMC9875935; doi:10.1002/hbm.26111)
Supplement: Supplementary file 1 — Appendix S1: [file HBM-44-914-s001.docx]

**Supplementary Materials**

Supplementary 1. The waveforms of each stimulation condition.


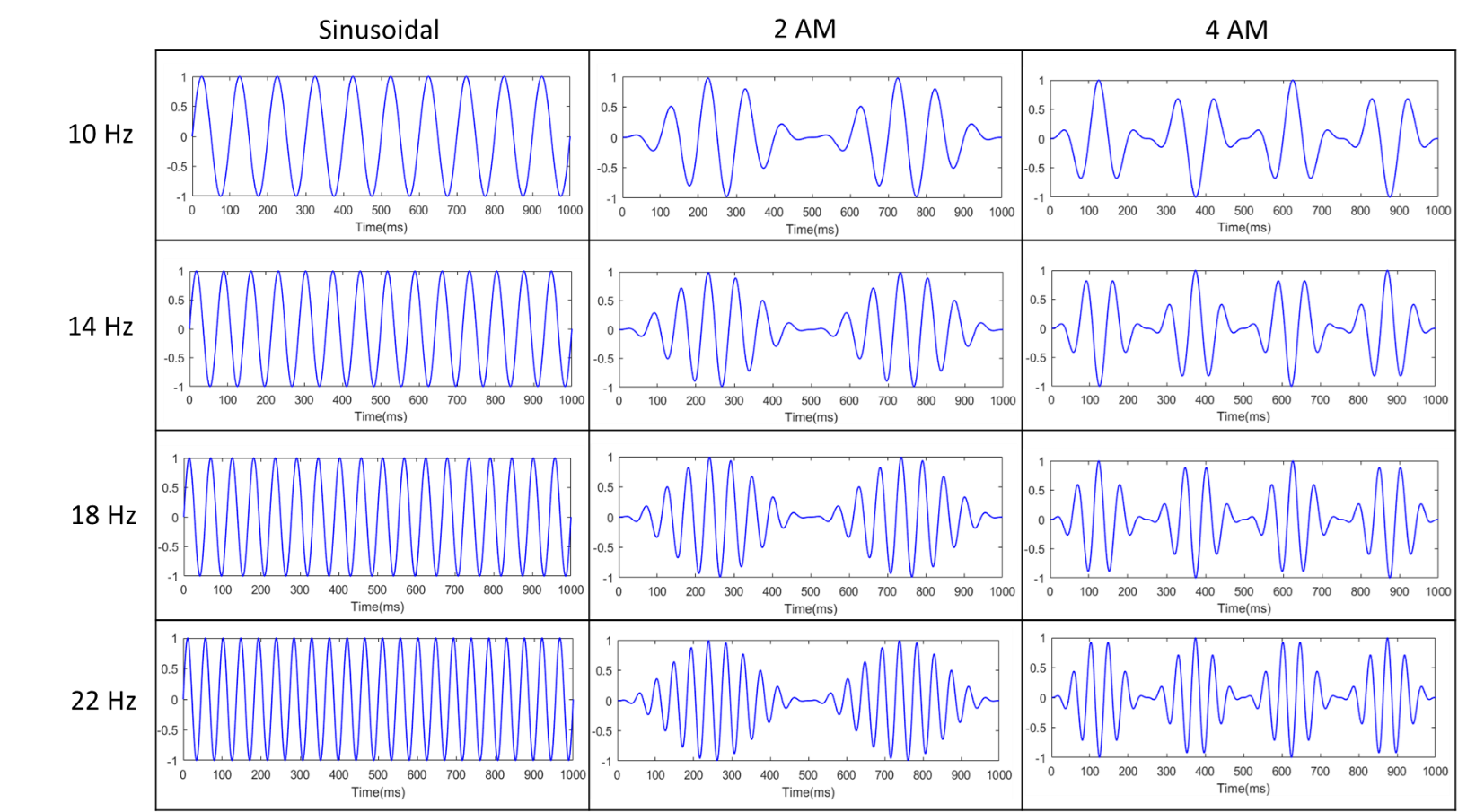


Figure S1. Illustrations of the given tACS waveforms. Waveforms in the same row have the same carrier frequency which is indicated on the left side of the table, and waveforms in the same column have the same AM frequency indicated on the upper side of the table.

Supplementary 2. Phosphene threshold result of the pilot study


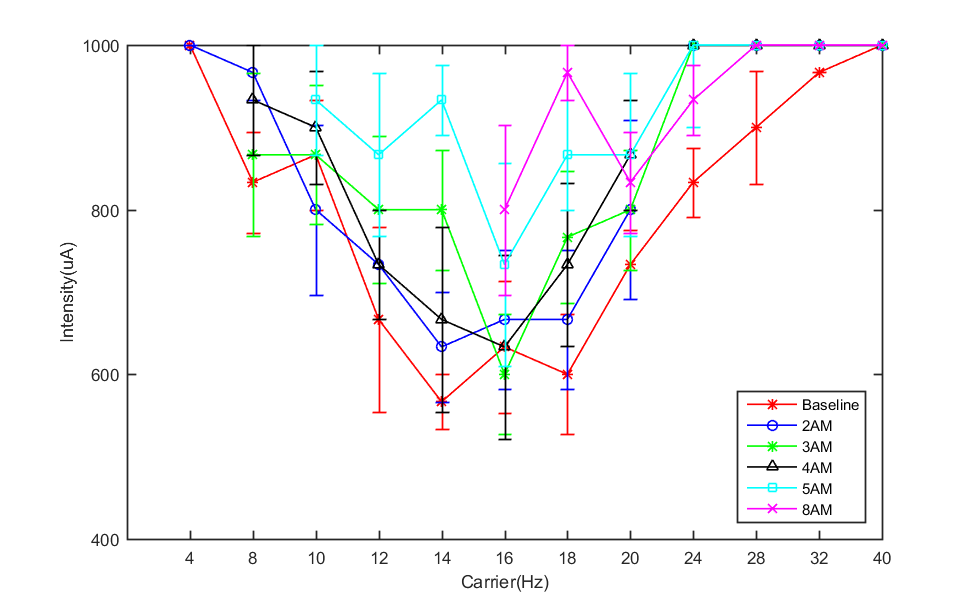


Figure S2. The pilot study recruited 6 healthy participants (3 females, mean age = 23±2.7). The experimental procedure contained only the threshold measurement using the MOBS (described in Supplementary 1). The carrier frequency ranged from 4 to 40 Hz, and the AM frequency ranged from 0 (baseline) to 8 Hz. As illustrated in Figure S1, the baseline (0 Hz AM) demonstrated the lowest threshold at 14 and 18 Hz, and no phosphene response (1000 uA) at 4 and 40 Hz. Moreover, the threshold increased as the AM frequency increased. To recruit a more visible response, we picked up the carrier frequency of 10, 14, 18, and 22 Hz, and the AM frequency of 0, 2, and 4 Hz.

Supplementary 3. The electrode correspondence in the current study and 10-10 system.

The following table lists the corresponding channel names of 10-10 system with the electrode numbers on the 128-channel HydroCel GSN based on the technical notes provided by Electrical Geodesics, Inc for electrodes adopted in our study. For EGI sensors without a corresponding location in 10-10 system, we describe the relative location of conventional 10-10 system channels. In the figures, sensors with outer frames of the same colors indicate they are in the same polarity. The sensors filled with yellow indicate an EGI sensor that also appears in 10-10 system.

Table S1. The corresponding list of EGI sensors and 10-10 system electrode names.

| Occipital montage | | Left facial montage | |
| --- | --- | --- | --- |
| 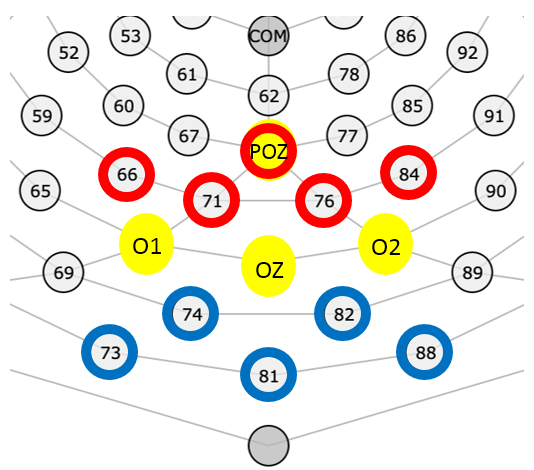 | | 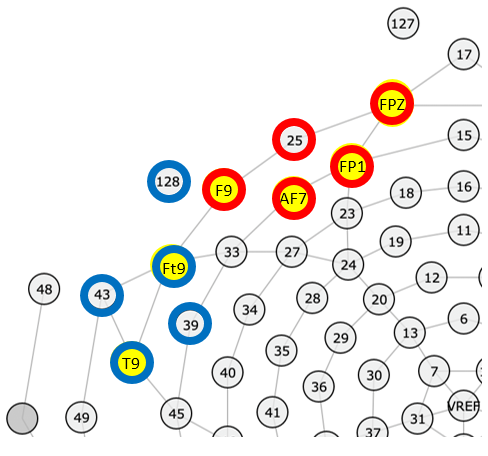 | |
| 128-channel GSN HydroCel | 10-10 system channel name | 128-channel GSN HydroCel | 10-10 system channel name |
| 66 |  | 38 | Ft9 |
| 71 |  | 39 |  |
| 72 | POZ | 43 |  |
| 76 |  | 44 | T9 |
| 84 |  | 128 |  |
| 73 |  | 21 | FPZ |
| 74 |  | 22 | FP1 |
| 81 |  | 25 |  |
| 82 |  | 26 | AF7 |
| 88 |  | 32 | F9 |
| Channels 66, 71, 76, 84 are below Po3, POZ, PO4, and channels 73, 74, 81, 82, 88 are below O1 Oz O2 channels of 10-10 EEG system. | | Channels 21, 22, 25, 26, 32 surround frontal channels, and 38, 39, 43, 44, 128 cover temporal channels corresponding 10-10 EEG system. | |

Supplementary 4. Phosphene threshold measurement

The measurement of phosphene threshold (PT) followed the procedures of the modified binary search (MOBS) method (Anderson & Johnson, 2006; Tyrrell and Owen, 1988). The initial lower and upper bounds were 500 and 1000 μA for the occipital stimulation and 0 and 300 μA for facial stimulation. For each frequency, the first intensity was the higher bound. For example, the first tested intensity was 1000 μA in the occipital stimulation condition. If the subject could not see phosphene, the threshold for the condition would be recorded as 1000μA and stopped testing. If the subject reported phosphene, the next intensity was the midpoint of the higher (1000 μA) and the lower (500 μA) bound, which was 750 μA. If phosphene were also reported with 750 μA intensity, 750 μA would become the new upper bound. The Midpoint of 750μA and the previous lower bound (500 μA) would serve as the next stimulation intensity, and so forth. On the other hand, if phosphene were not detected with 750 μA, 750 μA would become the new lower bound. Thus, the next intensity became the midpoint of 750 μA and the previous higher bound (1000 μA). The procedure was repeated until participants' responses were reversed from seen to unseen, and the intensity of the last seen trial was treated as the threshold intensity.

Supplementary 5. HHSA method and procedures

HHSA is a nonlinear analysis based on empirical mode decomposition (EMD, Huang et al., 1998). Our previous research has demonstrated that HHSA can reliably decompose nonlinear signals by representing the spectrum of carrier frequencies and AM frequencies in multi-dimensional representation (Juan et al., 2021; Liang et al., 2021; Nguyen et al., 2019). As illustrated in Figure S1, the raw signals were decomposed into several first-layer intrinsic mode functions (IMFs) with a masking EMD (Ensemble EMD, EEMD. Huang et al., 1998; Wu et al., 2009a, 2009b). EEMD was an enhanced algorithm of EMD that helped resolve the mode-mixing problem and reduce distortion of HHSA results.


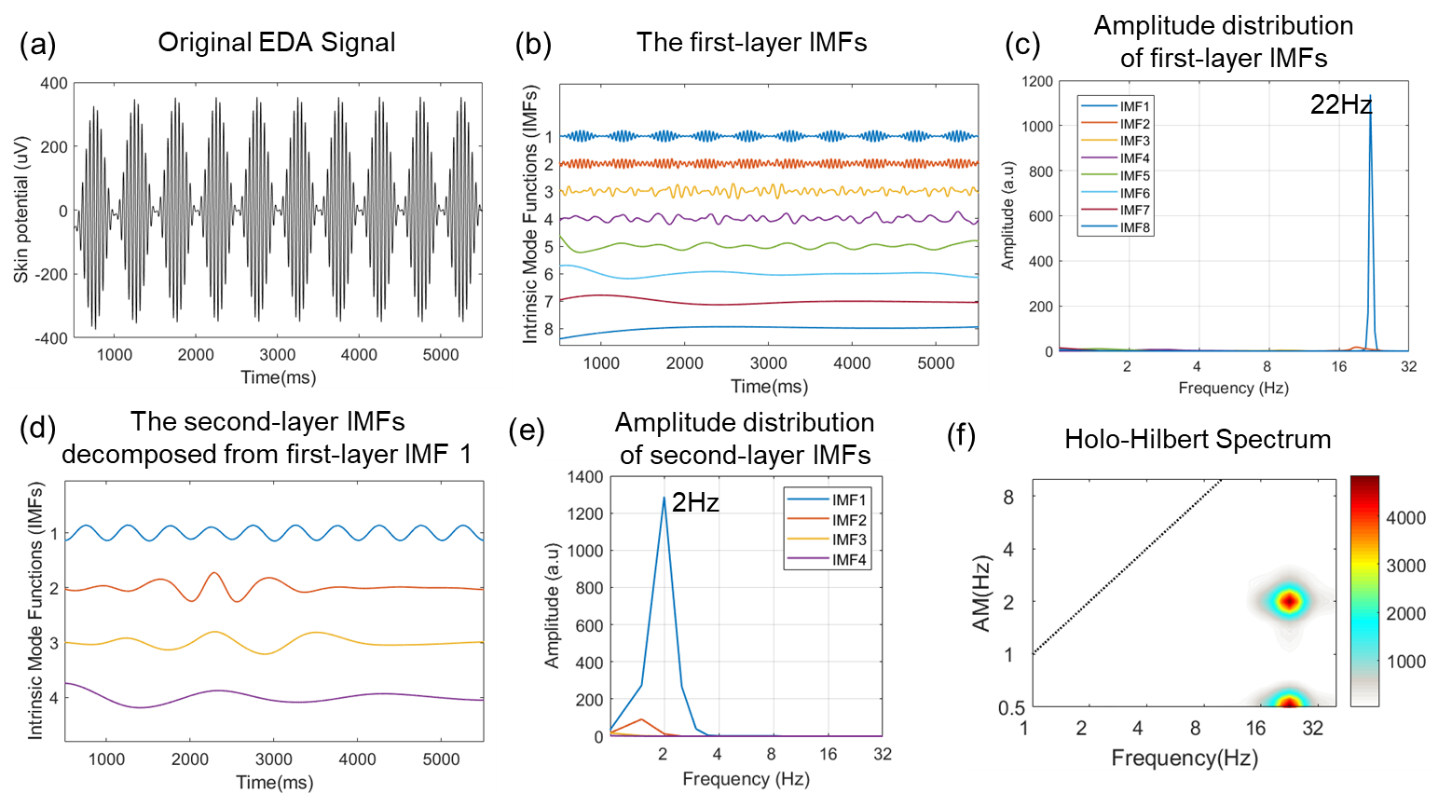


Figure S3. HHSA workflow of analyzing the near-eye EDA data collected under 2Hz AM 22Hz carrier stimulation. (a) illustrates the raw input data. (b) demonstrates the result of first layer IMFs decomposed with EEMD plotted in the normalized unit. The instantaneous frequency and amplitude information of each IMF were then extracted with Hilbert-Huang Transform (HHT, Huang et al., 1998). (c) demonstrates the amplitude distribution of first-layer IMFs across time. IMF1 had the greatest amplitude at 22Hz. The first-layer IMFs were then entered EEMD again for the AM frequency decomposition. (d) demonstrates the result of decomposing the first-layer IMF1. HHT on the second-layer IMFs revealed the greatest amplitude of 2Hz, as illustrated in (e). (f) illustrates the Holo-Hilbert spectrum calculated from the original signal in (a). The two-dimension spectrum comprises the normalized power of carrier frequency (X-axis) and AM frequency (Y-axis); both axes are in the dyadic scale. The highest energy focused on 22Hz carrier frequency and 2Hz AM frequency. This HHSA result reflects the cross-frequency combination relation in the original tACS signal.

The instantaneous frequencies and amplitudes were estimated from each first-layer IMF in each trial with the HHT. This step provided the time-frequency characteristic of the carrier frequency (*f_c_*) and corresponded to the conventional Fourier transform. The amplitude functions of the first-layer IMFs were then entered into the second-layer EMD to obtain the second-layer IMFs. Information of phase and amplitude was then estimated from the outputted second-layer IMFs to represent the time-frequency characteristics of amplitude modulation frequency (*f_am_*). Since the time factor was not considered in this analysis, we estimated the marginal sum of the carrier and AM frequencies power along the time dimension. Therefore, the illustrated power spectrum was a two-dimensional plot with a carrier frequency on the x-axis and AM frequency on the y-axis. The HHSA was executed with a customized MATLAB script with EEMD codes, as addressed in Liang et al. (Liang et al., 2021).

Because EMD served as a natural dyadic filter bank, the edges of frequency bands were represented by a formula of 2^n^ (Juan et al., 2021). Each dyadic frequency step was separated into eight sub-frequency bins (2^0.125^ for each step), and the power values were marginally summed from the $\pm1$ bin of the given frequency. For example, f_c_ = 14Hz fell in the frequency bin of 2^3.875^. Therefore, we summed the power values within 2^3.75^Hz –2^4^Hz (13.45Hz–16Hz) across time points on a linear scale. Based on this principle, the frequency ranges for each stimulation conditions are: 2Hz: 1.8-2.2Hz (2^0.875^-2^1.125^), 4Hz: 3.7-4.3Hz (2^1.875^-2^2.125^), 10Hz: 9.5-11.3Hz (2^3.25^-2^3.5^), 14Hz: 13.4-16Hz (2^3.75^-2^4^), 18Hz: 17.4-20.7Hz (2^4.125^-2^4.375^), and 22Hz: 20.7-24.7Hz (2^4.375^-2^4.625^). The summed powers were normalized by dividing the total power summed across all frequency bands and conditions to reduce between-participant variation. Mean powers were then calculated for each participant by averaging across conditions and conducting a paired t-test compared between left-face and occipital montages.

Supplementary 6. The phosphene drawings under threshold-intensity stimulation of all recruited participants.


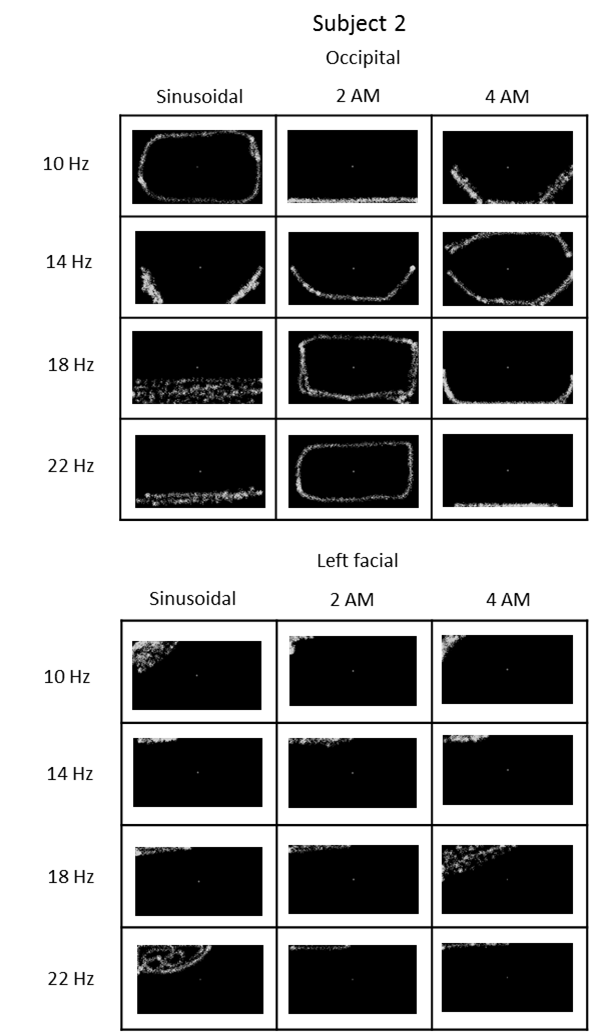

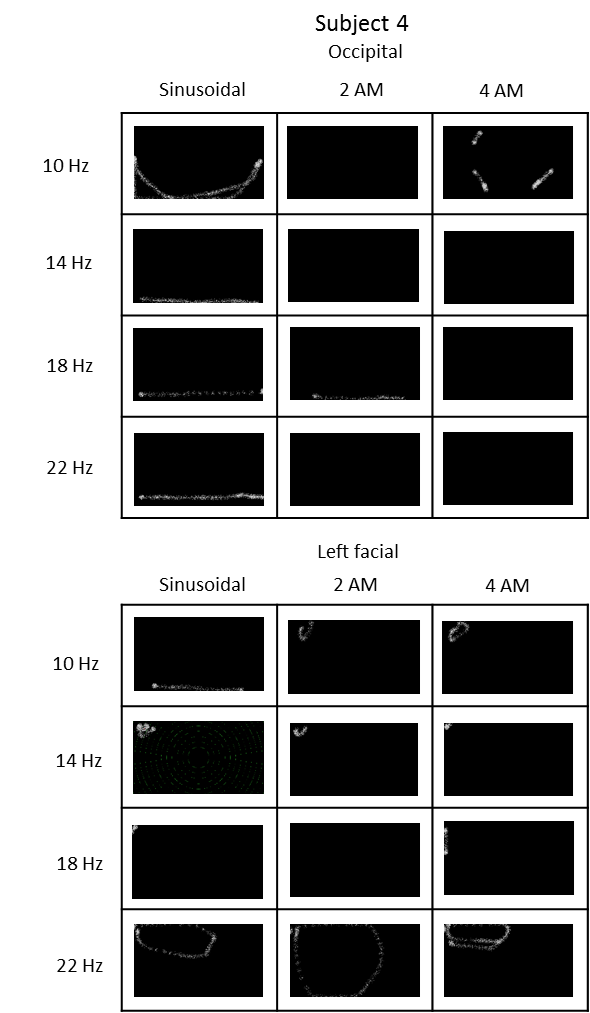


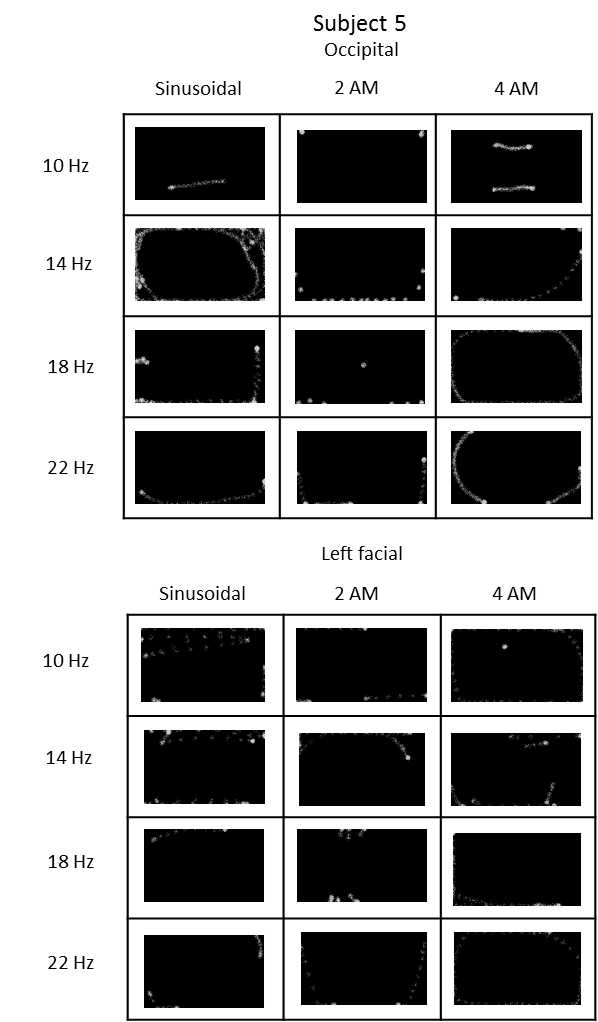

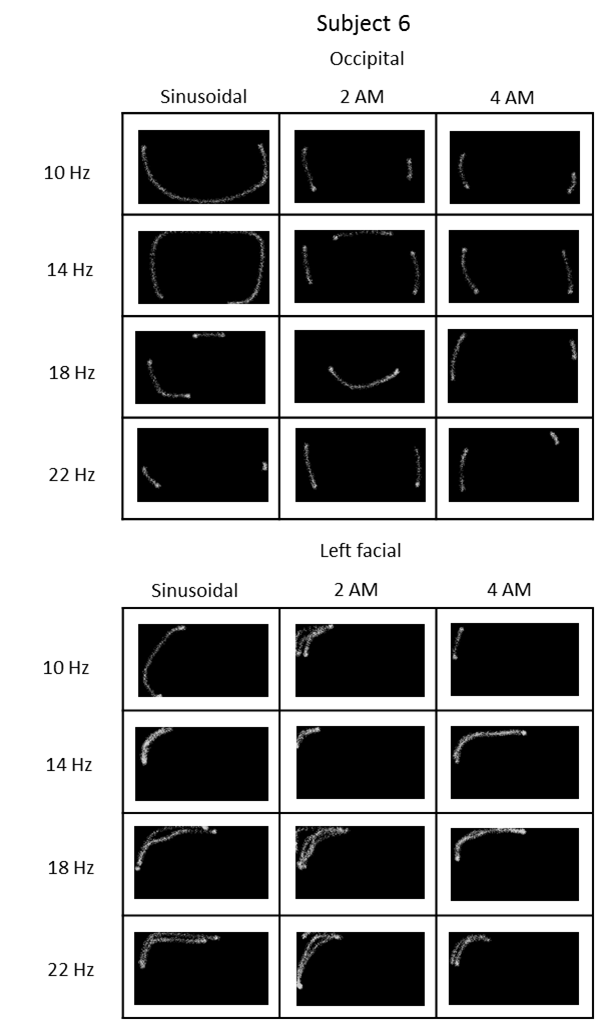


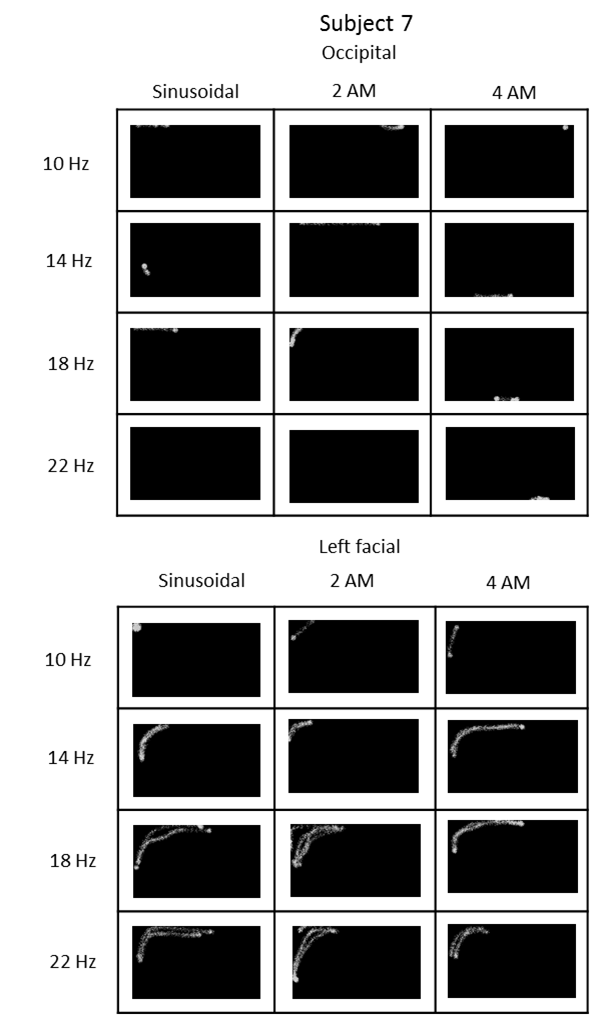

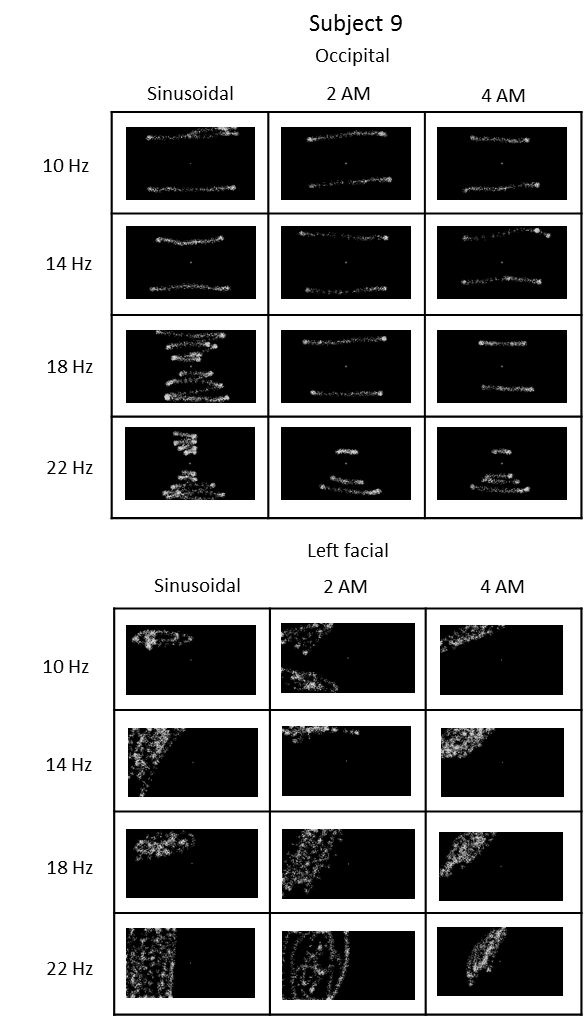


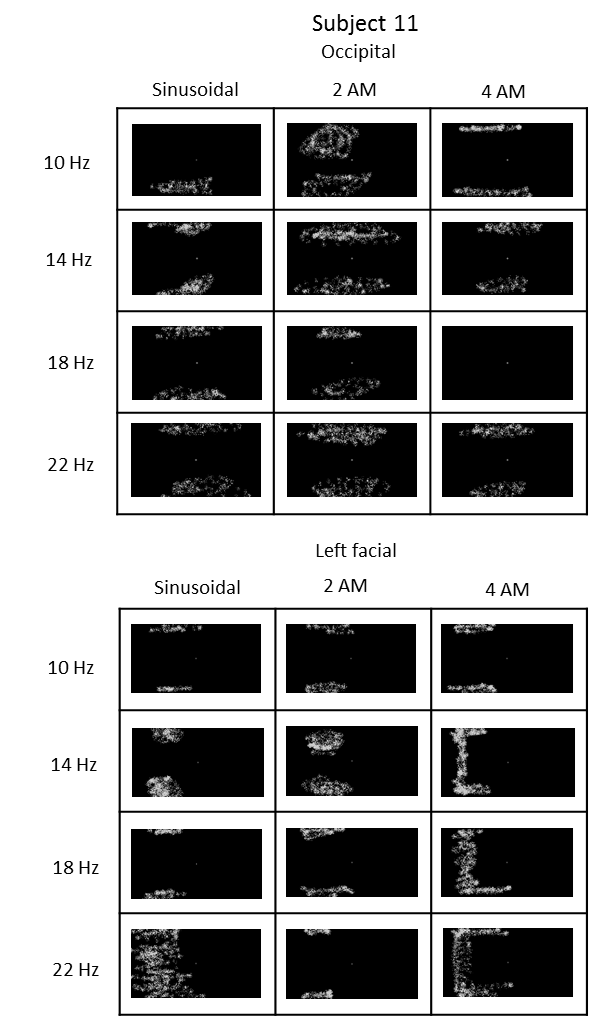

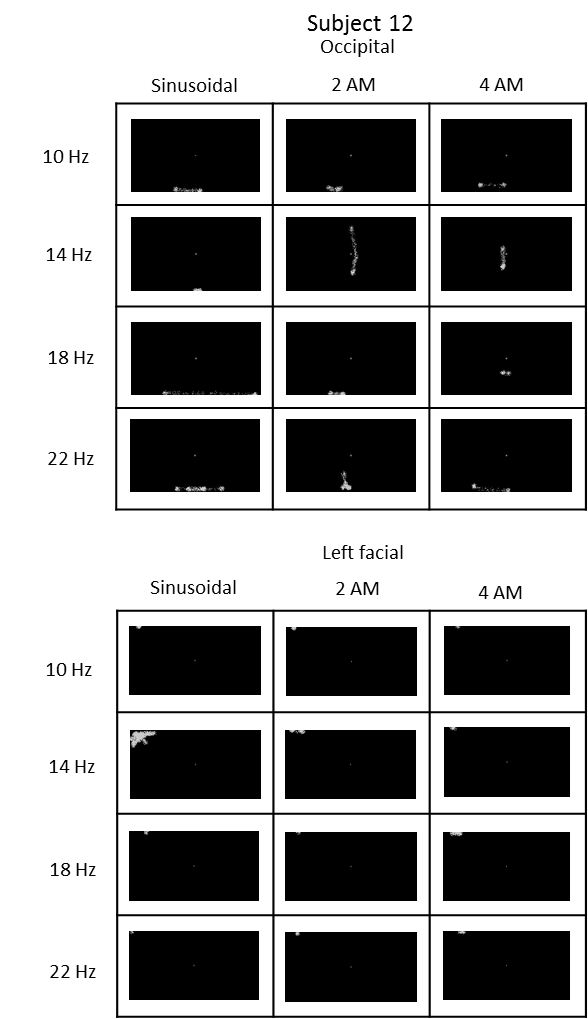


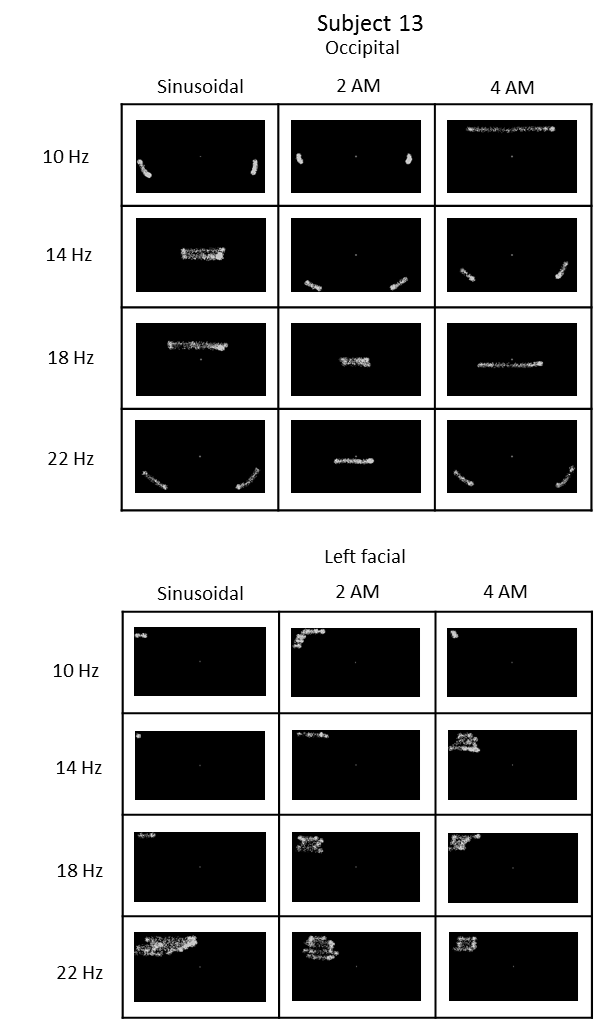

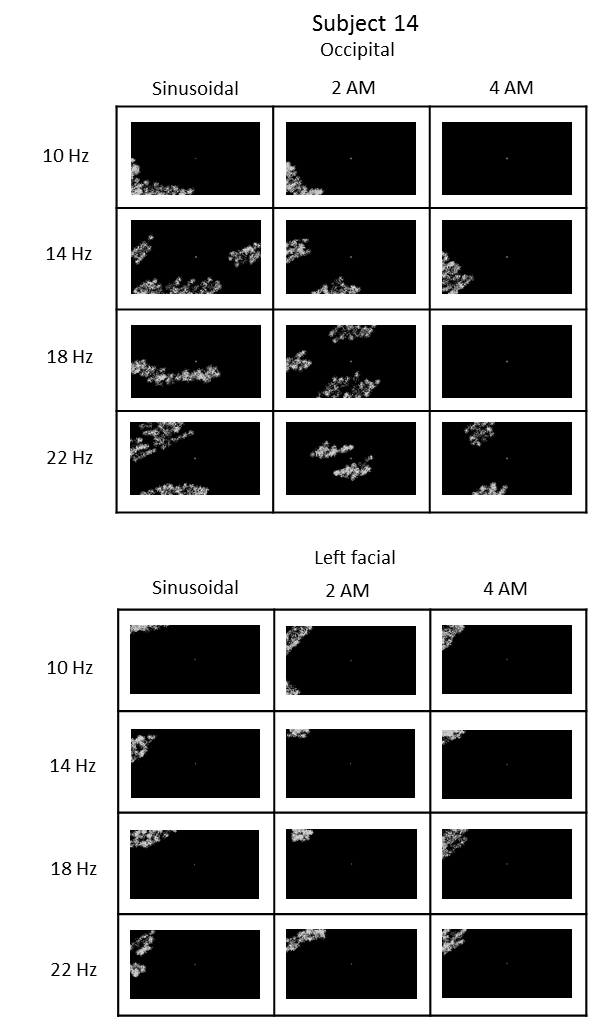

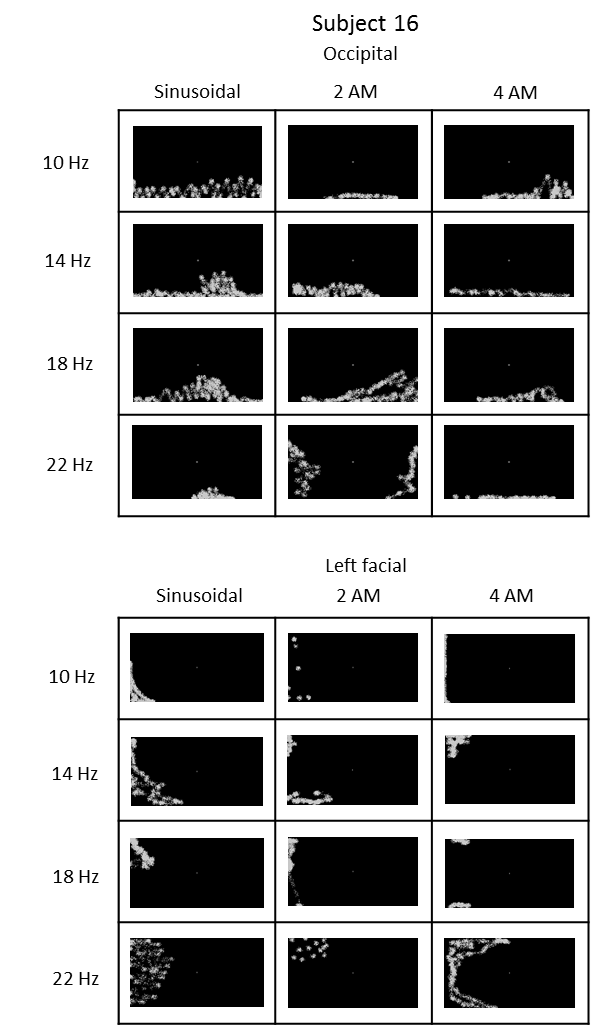


Supplementary7. The Holo-Hilbert spectrums of the mean power across 11 participants in all stimulation conditions and montages. The EDAs were all recorded under the “just above threshold intensity” stimulation. The x-axis indicates the carrier frequency, and the y-axis indicates the AM frequency. The power of carrier frequency appears at the bottom of the spectrum, located on the corresponding frequency of the x-axis. The power of AM is displayed at the intersection of the coupling carrier frequency and AM frequency. It should be noted that the scales of color bars were different between the occipital and left-facial montages that the power measured under occipital stimulation was significantly larger than which under left-facial stimulation.


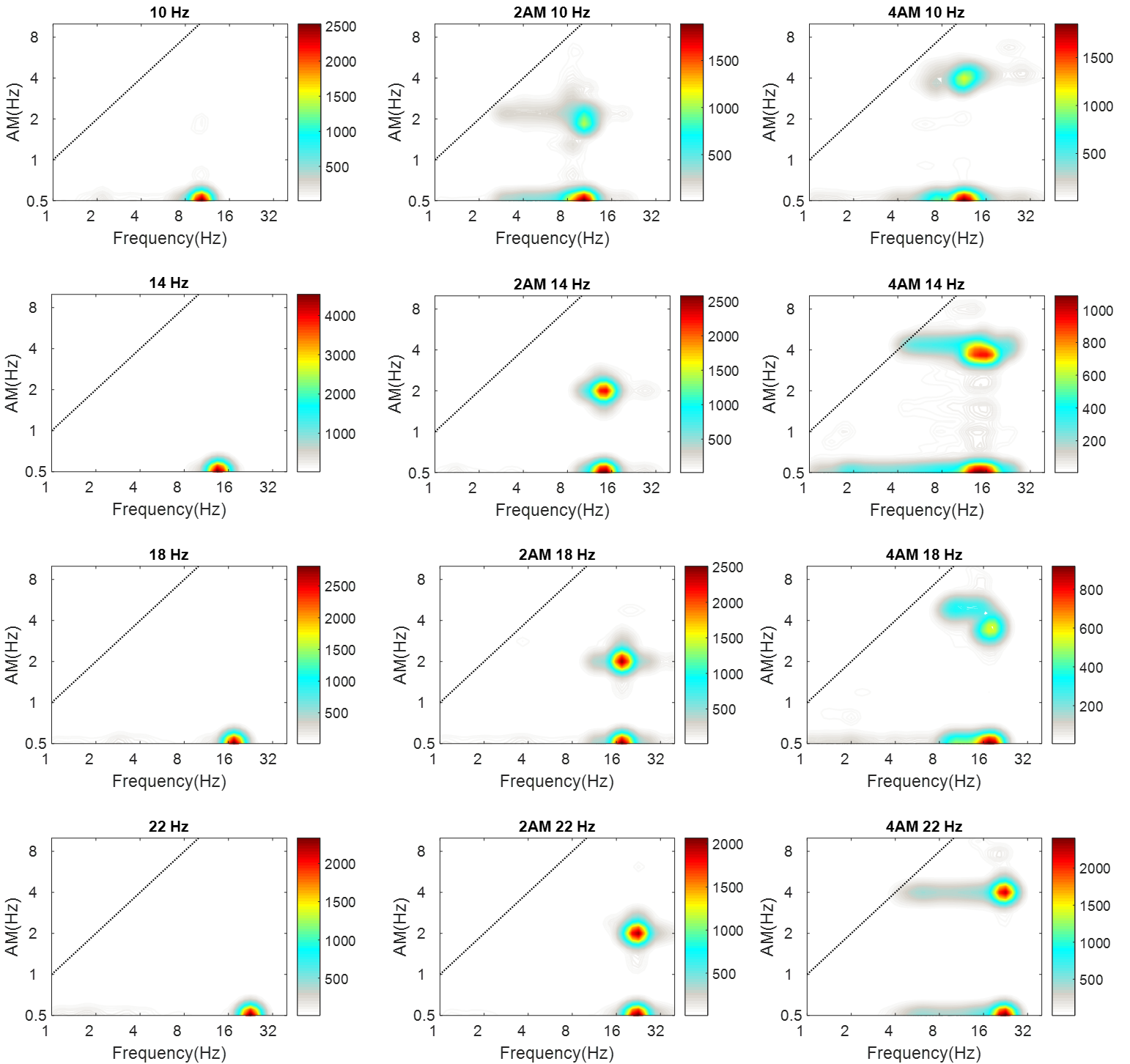


Figure S4. The Holo-Hilbert spectrum represents the power distributions of EDA signals recorded under occipital stimulation.


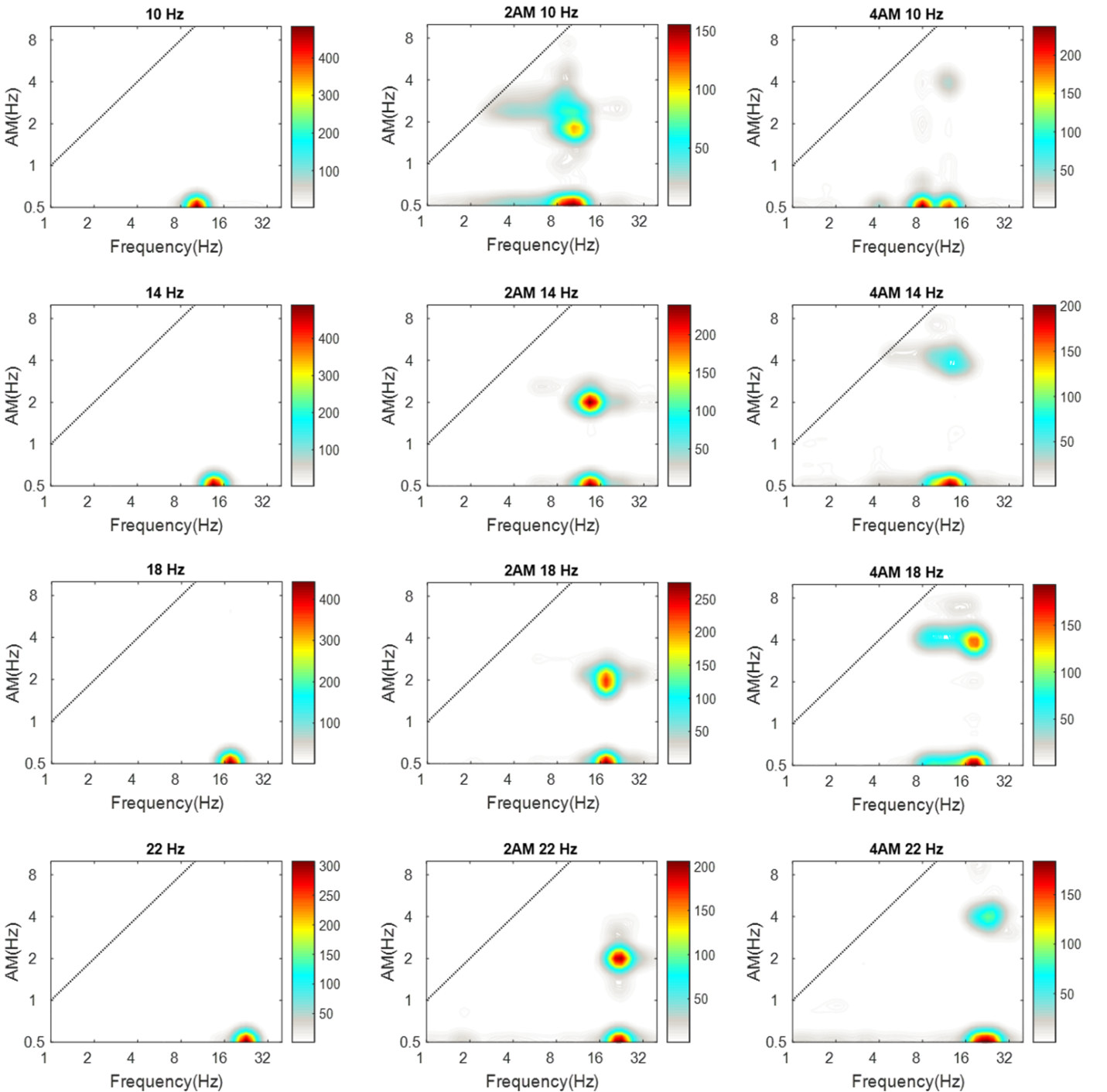


Figure S5. The Holo-Hilbert spectrum represents the power distributions of EDA signals recorded under left-facial stimulation.

Supplementary 8. Further analysis examining the EDA power under equal stimulation intensity.


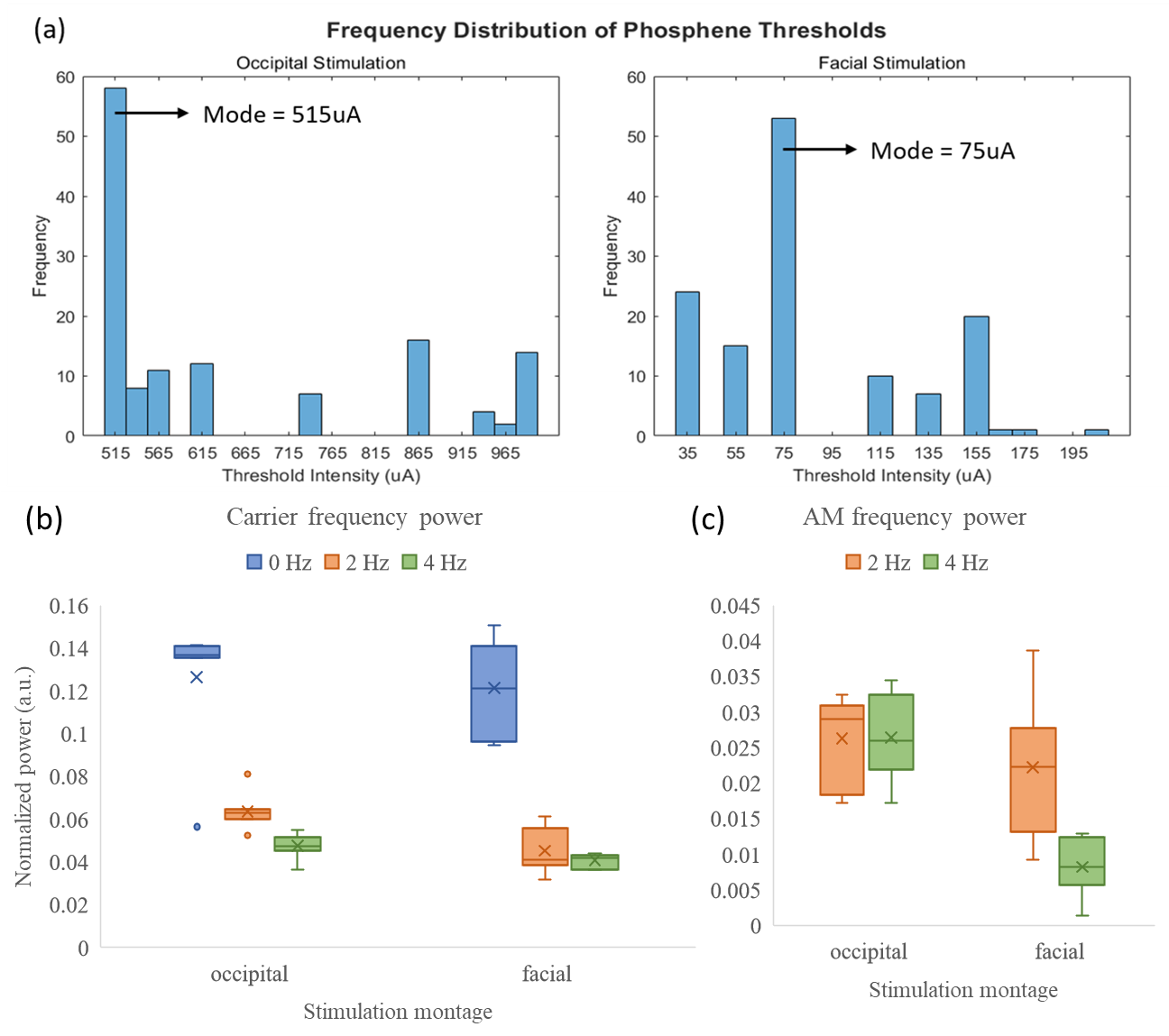


Figure S6. (a) The frequency distribution of phosphene threshold intensities in the occipital stimulation (left) and left-facial stimulation (right). As can be seen from the bar graph, the most frequent threshold intensity was 515 μA in occipital stimulation, and 75 μA in left-facial stimulation. We, therefore, selected the near-eye EDA powers in trials under these intensities for analysis. We firstly averaged the power values across carrier frequencies within each participant. Each participant would therefore have 3 mean power values (corresponding to 0 Hz, 2Hz, and 4 Hz AM) in the carrier frequency power, and 2 mean power values (corresponding to 2 Hz and 4 Hz AM, no numbers for 0 Hz AM) in the AM frequency power. Since the EDA was only recorded under the threshold intensity, participants whose threshold did not fall into these numbers would not have an EDA record and became missing values. To deal with the missing values, we exclude participants who had more than one missing value in each montage and then filled the rest of the missing values with the across-subject mean in the same condition. Following this processing, a total of 7 participants remained for analysis. Two repeated measured ANOVA employed factors of montage (occipital vs. facial) and AM frequency (0 Hz, 2 Hz, 4 Hz) were conducted to the carrier frequency power and AM frequency power respectively.

The results are illustrated in (b) and (c). (b) The boxplot of carrier frequency power. The ANOVA result on the carrier frequency power demonstrated significant AM frequency effect (*F*_(2,12)_ = 97.49, *p <* 0.0001, η_p_^2^ = 0.94, ε = 0.55), while no montage effect (*p* = 0.06) or interaction between montage and AM frequency (*p* = 0.5) was found. Post hoc analysis with Bonferroni correction revealed that the power was greater for 0 Hz AM (0.124±0.018) than for 2 Hz AM (0.054±0.006) or 4 Hz AM (0.044±0.003). Also, the power was greater for 2 Hz than 4 Hz. (c) The boxplot of AM frequency power. ANOVA conducted on the AM frequency power showed main effect of montage (*F*_(1,6)_ =30.19, *p =* 0.002, η_p_^2^ = 0.83), AM frequency (*F*_(1,6)_ =9.91, *p =* 0.02, η_p_^2^ = 0.62), and the interaction between montage and AM frequency (*F*_(1,6)_ =30.16, *p =* 0.002, η_p_^2^ = 0.83). Main effect of montage revealed greater power for occipital stimulation (0.026±0.005) than for facial montage (0.015±0.005). Main effect of AM frequency demonstrated greater power for 2 Hz AM (0.024±0.006) than for 4 Hz AM (0.017±0.004). The post hoc comparisons on the interaction showed that the AM frequency effect was significantly found only in the facial stimulation montage (*F*_(1,6)_ =21.29, *p =* 0.004, η_p_^2^ = 0.78), but not in the occipital montage (*F*_(1,6)_ =0.004, *p =* 0.95, η_p_^2^ = 0.001).

It can be concluded from the results that (1) the difference in the threshold intensity between AM (2 Hz and 4 Hz) and non-AM (0 Hz) conditions is a result of less power delivery in AM conditions. (2) We also found a power difference between 2 Hz and 4 Hz AM conditions, which may explain the threshold difference between these two AM frequency conditions in occipital stimulation.

Supplementary 9. Computational simulation of cortical electric field

In this analysis, we conducted cortical electric field simulation with the open-source toolbox ROAST (Huang et al., 2019) on a subset of 8 out of 12 participants who provided individual T1 structure images. ROAST is a toolbox that simulated the current and electric field based on transcranial direct current stimulation (tDCS). Alternating current from tACS is considered to reach quasi-static and will receive the same result (Grossman et al., 2017; Neuromodec, 2020). Therefore, we conducted only one simulation for each PT intensity regardless of its frequency condition. The electrodes are identical to the stimulation electrodes described in the method session. The results of the simulation are illustrated in Figures S7 and S8.


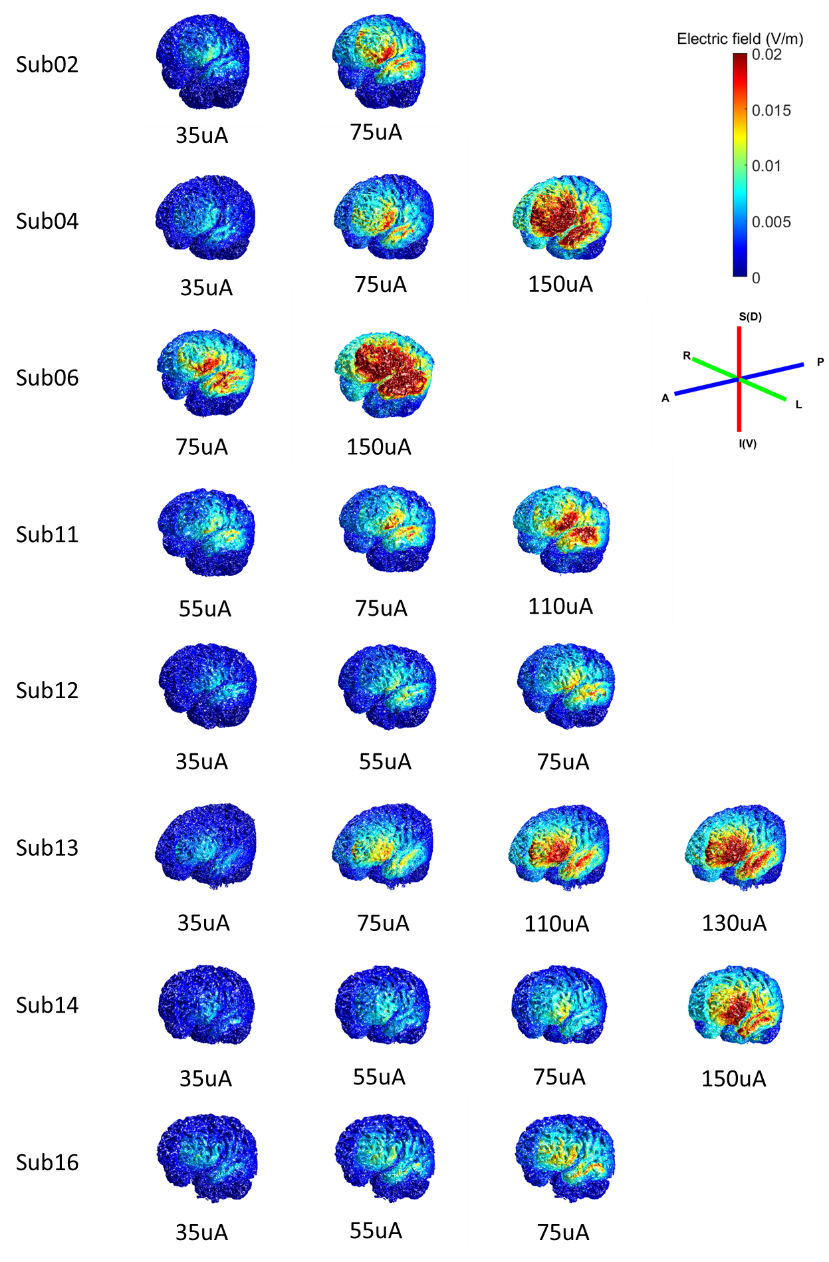


Figure S7. ROAST simulation results of left facial montage stimulation. Simulations from the same participant are illustrated in the same row, with the stimulation intensity underneath. The electric fields focused on left frontal to temporal cortical area and changed as a function of the given intensity.


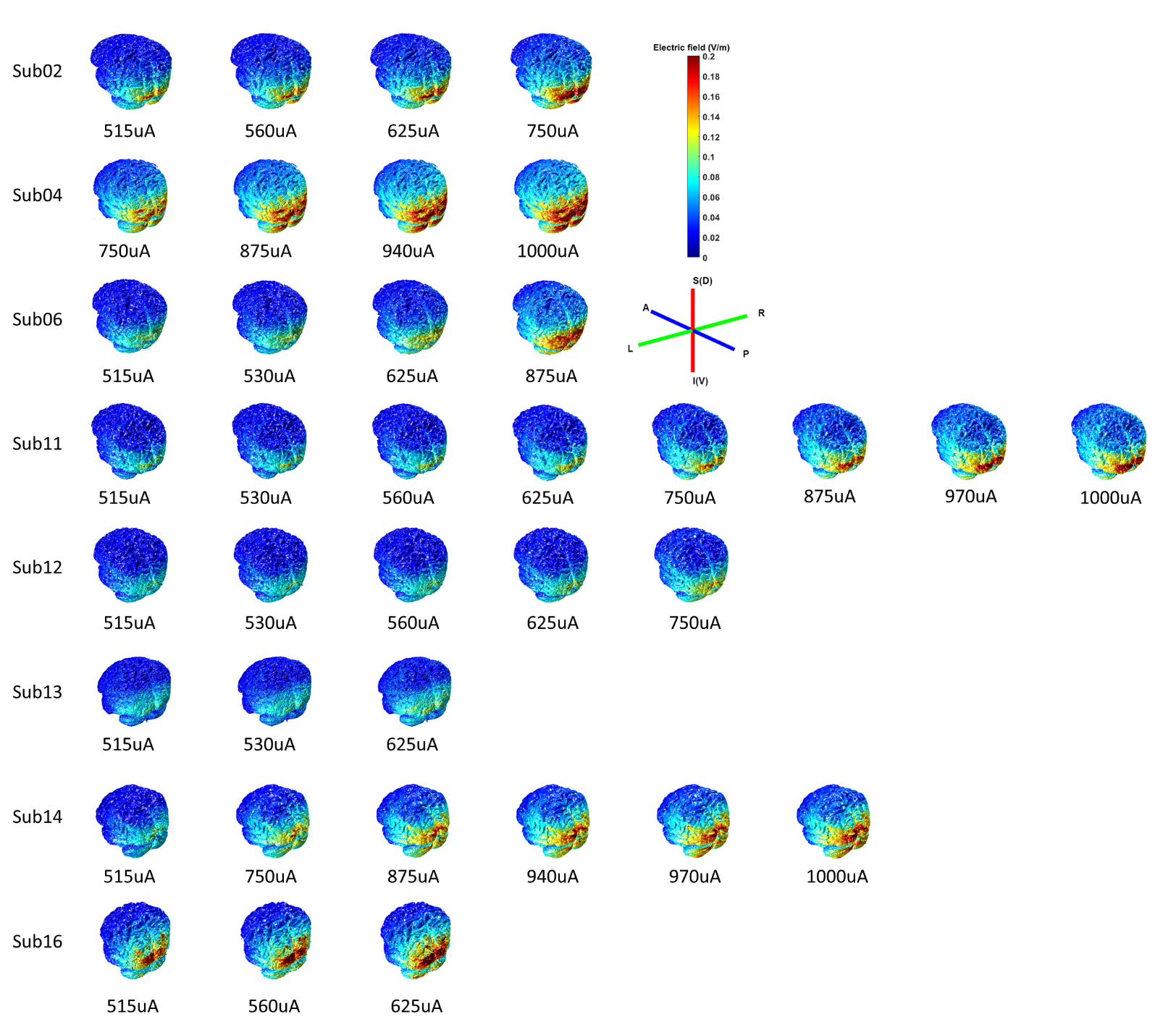


Figure S8. ROAST simulation results of occipital stimulation. Simulations from the same participant are illustrated in the same row, with the stimulation intensity underneath. The electric fields focused on the central visual cortical area and changed as a function of the given intensity.

The output results were electric field magnitude with a unit of V/m. The magnitude of the electric field was extracted from 4 regions on the tip of the bilateral visual cortex (28, -96, -6 and -28, -96, -6; illustrated in Figure S9a) and the left anterior and orbital frontal cortex (-11, 38, -19 and -23, 55, 4, respectively; illustrated in Figure S9b), where these regions are considered to be close to the eye. Values from the bilateral visual cortex were averaged into “posterior electric field value (posterior EF)” and the values from the left anterior and orbital frontal cortex were averaged into “anterior electric field value (anterior EF).” Based on the type of stimulation montage and position of EF, we gathered the data into four data sets: Occipital Montage-Anterior EF, Occipital montage-Posterior EF, Left facial montage-Anterior EF and Left facial montage-Posterior EF. The Shapiro-Wilk normality tests conducted on the four data sets indicated that the distribution of these data sets did not fulfill normal distribution (Figure S10), and thus we conducted nonparametric analyses on the data.


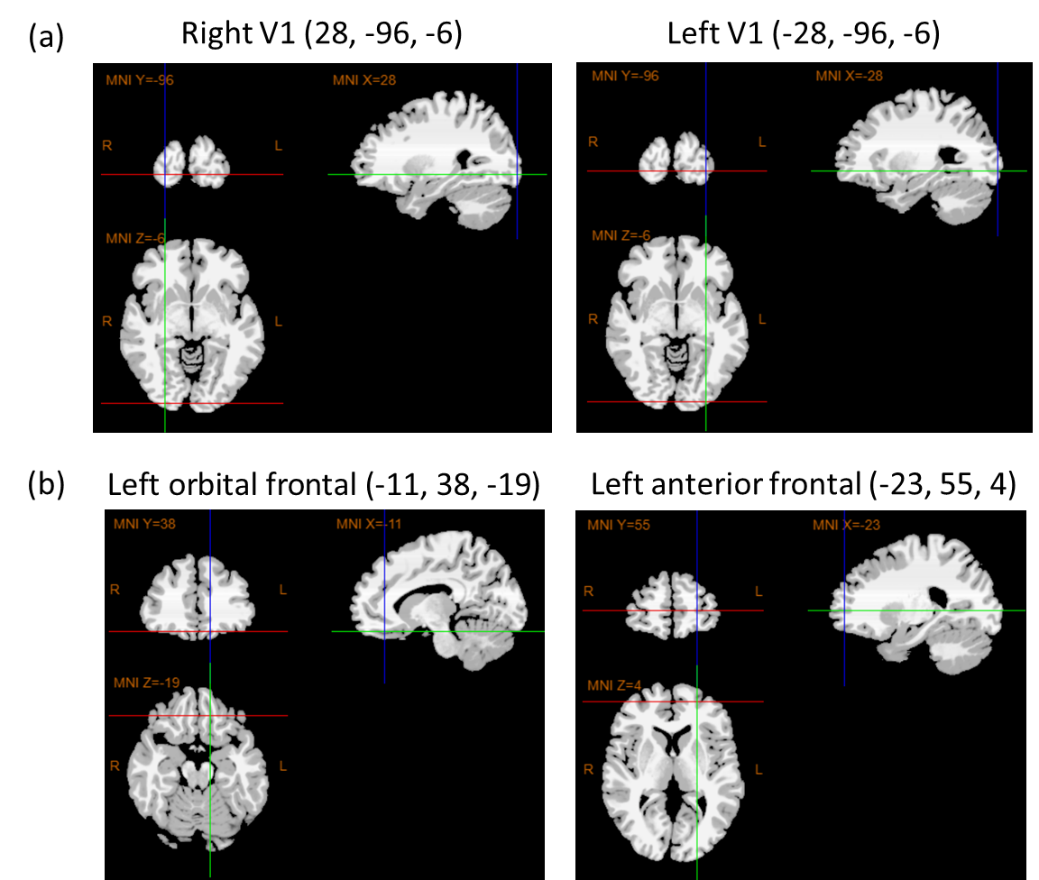


Figure S9. MNI coordinates and locations where electric field magnitude values were extracted from. (a) posterior electric field values were extracted from bilateral visual areas (28, -96, -6 and -28, -96, -6) under the stimulated area. (b) anterior electric fields were extracted from the left anterior (-11, 38, -19) and orbital frontal cortex (-23, 55, 4) where these areas are thought to be close to the orbit.

Figure S10. The data normality test and QQ plot for each set of the data. Upper left: Anterior EF under occipital stimulation, Upper right: Posterior EF under occipital stimulation, Lower left: Anterior EF under left facial stimulation, and Lower right: Posterior EF under left facial stimulation.

The estimated EF values of each trial are illustrated in violin plots in Figure S11a. We first examined whether the estimated EF decreased with the distance from the stimulated area. We predicted that the posterior EF should be greater than the anterior EF in the occipital montage, and smaller than the anterior EF in the left facial montage. Two Mann-Whitney U tests were performed on EF values under occipital and left-facial montages respectively. As illustrated in Figure S11a, the statistical results followed our prediction. The EFs were higher for the posterior than anterior EF during occipital stimulation (Z = -11.97, p<0.001) and higher for anterior than posterior EF during left-facial stimulation (Z = 11.27, p<0.001). We then performed Kendall correlations to examine the relation between the given stimulation intensity and the estimated EF magnitude. The results in Figure S9b showed that in all EF data sets, the estimated EF values were positively correlated with the intensity of the given stimulation. Our analyses of the estimated cortical EF values reveal that the cortical electrical field well reflected the current intensity given outside the scalp. However, it is impossible to differentiate the frequency effect by the estimation due to the limitation of computational simulation.


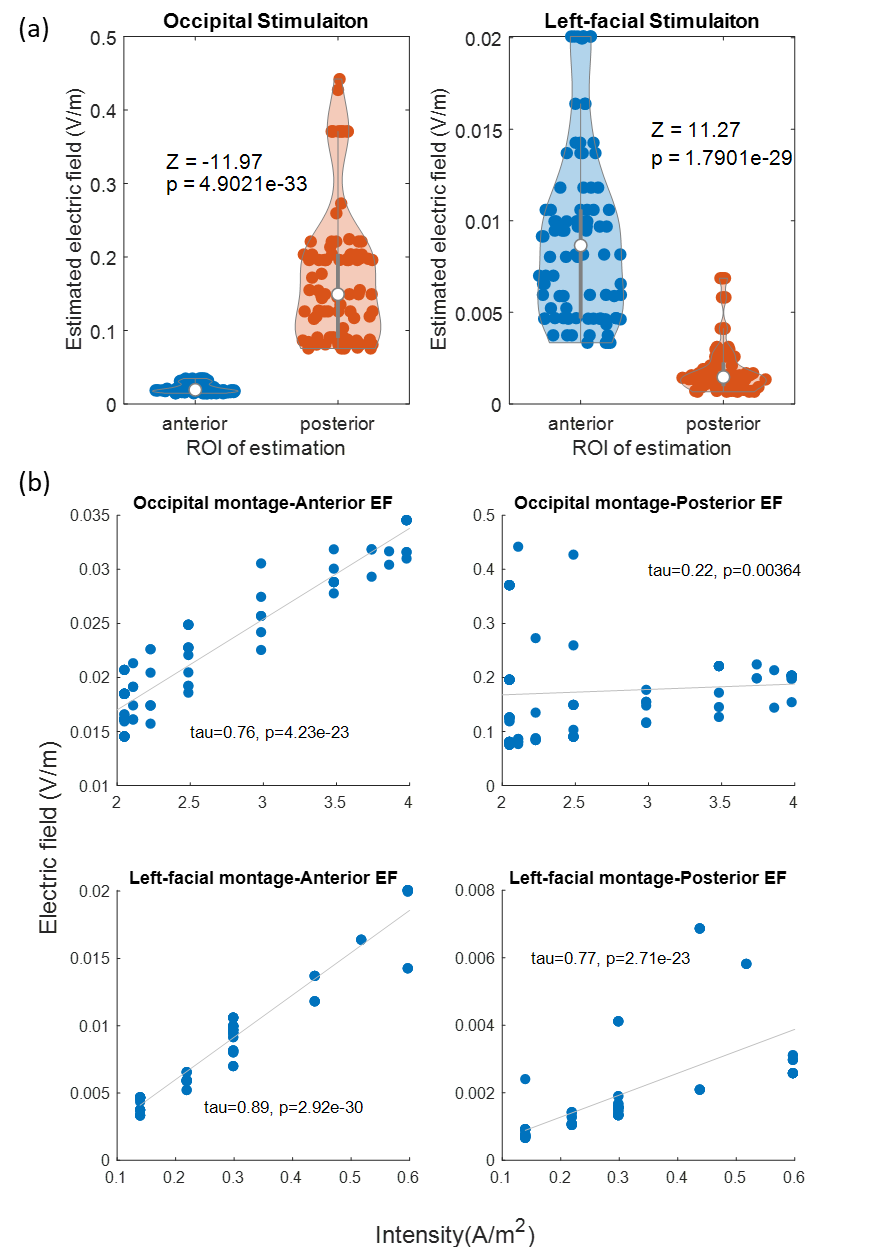


Figure S11. (a) Violine plots of the estimated EF from anterior and posterior cortex areas under occipital and left-facial stimulation montages. The posterior EF value (orange dots) was stronger than anterior EF (blue dots) in the occipital stimulation (left) but weaker in the facial stimulation (right). (b) The scatterplots indicate relationships between given intensity (x-axis) and estimated cortical EF values (y-axis) on the frontal cortex (upper left) and visual cortex (upper right) under occipital stimulation, and on the frontal cortex (lower left) and visual cortex (lower right) under left-facial stimulation. Separated Kendall’s correlation tests conducted on these four data sets demonstrated significant positive correlations in all datasets, indicating that the higher intensity given outside the scalp, the cortical EF was stronger in response.

References

Anderson AJ, Johnson CA. Comparison of the ASA, MOBS, and ZEST threshold methods. *Vision Res*. 2006;46(15):2403-2411. doi:10.1016/j.visres.2006.01.018

Grossman N, Bono D, Dedic N, et al. Noninvasive Deep Brain Stimulation via Temporally Interfering Electric Fields. *Cell*. 2017;169(6):1029-1041.e16. doi:10.1016/j.cell.2017.05.024

Huang, Y., Datta, A., Bikson, M., Parra, L.C., Realistic vOlumetric-Approach to Simulate Transcranial Electric Stimulation -- ROAST -- a fully automated open-source pipeline, Journal of Neural Engineering, Vol. 16, No. 5, 2019.

Huang, N. E., Shen, Z., Long, S. R., Wu, M. C., Shih, H. H., Zheng, Q., Yen, N.-C., Tung, C. C., & Liu, H. H. The Empirical Mode Decomposition and the Hilbert Spectrum for Nonlinear and Non-Stationary Time Series Analysis. *Proc. Math. Phys. Eng. Sci.*1998:454(1971), 903–995. <http://www.jstor.org/stable/53161>

Juan CH, Nguyen KT, Liang WK, et al. Revealing the Dynamic Nature of Amplitude Modulated Neural Entrainment with Holo-Hilbert Spectral Analysis. *Front Neurosci*. 2021;15:673369. Published 2021 Aug 5. doi:10.3389/fnins.2021.673369

Liang WK, Tseng P, Yeh JR, Huang NE, Juan CH. Frontoparietal Beta Amplitude Modulation and its Interareal Cross-frequency Coupling in Visual Working Memory. *Neuroscience*. 2021;460:69-87. doi:10.1016/j.neuroscience.2021.02.013

Neuromodec. *ROAST: TES modeling made easy | Session 4 | NYC Neuromodulation 2020 Online Conference*. (2020, May 4). [Video]. YouTube.

Nguyen KT, Liang WK, Lee V, et al. Unraveling nonlinear electrophysiologic processes in the human visual system with full dimension spectral analysis. *Sci Rep*. 2019;9(1):16919. Published 2019 Nov 15. doi:10.1038/s41598-019-53286-z

Tyrrell, R.A., Owens, D.A. A rapid technique to assess the resting states of the eyes and other threshold phenomena: The Modified Binary Search (MOBS).*Behavior Research Methods, Instruments, & Computers* 1988;20:137–141. <https://doi.org/10.3758/BF03203817>

Wu, Z., Huang, N.E. Ensemble Empirical Mode Decomposition: A Noise-Assisted Data Analysis Method. *Adv Adapt Data Anal.* 2009;1(01):1–41. <https://doi.org/10.1142/S1793536909000047>

Wu, Z., Huang, N.E., Chen, X. The Multi-Dimensional Ensemble Empirical Mode Decomposition Method. *Adv Adapt Data Anal* 2009;1(03):339–372. <https://doi.org/10.1142/S1793536909000187>
